# Neuromuscular Electrical Stimulation for Intermittent Claudication (NESIC): multicentre, randomized controlled trial

**DOI:** 10.1093/bjs/znad299

**Published:** 2023-09-25

**Authors:** Laura Burgess, Adarsh Babber, Joseph Shalhoub, Sasha Smith, Consuelo N de la Rosa, Francesca Fiorentino, Bruce Braithwaite, Ian C Chetter, James Coulston, Manjit S Gohel, Robert Hinchliffe, Gerard Stansby, Alun H Davies, for the NESIC Trial Investigators, M S Gohel, A Pentelow, P Shipley-Cribb, R Elliot, N Nacorda, R Ward, D Read, A H Davies, J Shalhoub, T Lane, L Bolton, T V Le-Magowan, L Burgess, B Jones, N Strevens, A M Malagoni, S Tavares, A Henry, C Connelly, J Smee, R Toledano, J Nunag, L Tarusan, N Yasmin, C Carr, J Metcalfe, B Page, S Williams, D Hill, G Belt, A Rees, S Palmer, S Horton, D Lovelock, G Stansby, N Parr, M Catterson, E Scott, L Wales, J McCaslin, M Clarke, S Kirkup, D Amis, A Robinson, A Phillipson, S Covill, V Wealleans, E Fairbairn, I Chetter, A Harwood, J Long, J Totty, A Mohamed, T Wallace, J Hatfield, P Cai, S Pymer, J Palmer, A Firth, T Roe, S Ibeggazene, L Andrews, J Coulston, A Stewart, K Roberts, J Rewbury, S Mitchell, H Mills, L Vickery, C Adams, S Shakya, R Hadley, L Timewell, C Williams, J Kanapathipillai, J Hutter, F Goodchild, N Greig, J Blackall, K O’Callaghan, J Lucas, B Braithwaite, R Simpson, R Hadley, D Rittoo, C Thomson, L Vamplew, M Letts, T Webb, E Howe, A Fraine, J Kelly, F Beecham, N Pal, M Hulse, P Patel, I Nordon, S Smith, F Smith, H Yates, C Boxall, J Harvey, S Hammond, R Hinchliffe, H Cheshire, K Harding, S McIntosh, L Poole, P Brock, P Holt, N Sachsinger, R Ingham, J Budge, J Pang, P Ribeiro

**Affiliations:** Department of Surgery and Cancer, Imperial College London, London, UK; Imperial Vascular Unit, Imperial College Healthcare NHS Trust, London, UK; Department of Surgery and Cancer, Imperial College London, London, UK; Imperial Vascular Unit, Imperial College Healthcare NHS Trust, London, UK; Department of Surgery and Cancer, Imperial College London, London, UK; Imperial Vascular Unit, Imperial College Healthcare NHS Trust, London, UK; Department of Surgery and Cancer, Imperial College London, London, UK; Imperial Vascular Unit, Imperial College Healthcare NHS Trust, London, UK; Imperial Clinical Trials Unit, Imperial College London, London, UK; Department of Surgery and Cancer, Imperial College London, London, UK; Imperial Clinical Trials Unit, Imperial College London, London, UK; Nightingale-Saunders Clinical Trials & Epidemiology Unit (King’s Clinical Trials Unit), King’s College London, London, UK; One Stop Vascular Clinic, Queen’s Medical Centre, Nottingham University Hospitals NHS Trust, Nottingham, UK; Academic Vascular Surgical Unit, Hull York Medical School, University of Hull/Hull University Teaching Hospital NHS Trust, Hull, UK; Department of Vascular Surgery, Somerset NHS Foundation Trust, Taunton, UK; Department of Vascular Surgery, Cambridge University Hospitals NHS Foundation Trust, & NIHR Cambridge Biomedical Research Centre, Cambridge, UK; Department of Vascular Surgery, North Bristol NHS Trust, Bristol, UK; Northern Vascular Unit, The Newcastle Upon Tyne Hospitals NHS Foundation Trust, Newcastle, UK; Department of Surgery and Cancer, Imperial College London, London, UK; Imperial Vascular Unit, Imperial College Healthcare NHS Trust, London, UK

## Abstract

**Methods:**

This was an open, multicentre, randomized controlled trial. Patients with intermittent claudication attending vascular surgery outpatient clinics were randomized (1:1) to receive either neuromuscular electrical stimulation (NMES) or not in addition to local standard care available at study centres (best medical therapy alone or plus supervised exercise therapy (SET)). The objective of this trial was to investigate the clinical efficacy of an NMES device in addition to local standard care in improving walking distances in patients with claudication. The primary outcome was change in absolute walking distance, measured by a standardized treadmill test at 3 months. Secondary outcomes included intermittent claudication (IC) distance, adherence, quality of life, and haemodynamic changes.

**Results:**

Of 200 participants randomized, 160 were included in the primary analysis (intention to treat, Tobit regression model). The square root of absolute walking distance was analysed (due to a right-skewed distribution) and, although adjunctive NMES improved it at 3 months, no statistically significant effect was observed. SET as local standard care seemed to improve distance compared to best medical therapy at 3 months (3.29 units; 95 per cent c.i., 1.77 to 4.82; *P* < 0.001). Adjunctive NMES improved distance in mild claudication (2.88 units; 95 per cent c.i., 0.51 to 5.25; *P* = 0.02) compared to local standard care at 3 months. No serious adverse events relating to the device were reported.

**Conclusion:**

Supervised exercise therapy is effective and NMES may provide further benefit in mild IC.

This trial was supported by a grant from the Efficacy and Mechanism Evaluation Program, a Medical Research Council and National Institute for Health and Care Research partnership. Trial registration: ISRCTN18242823.

## Teaser

Supervised exercise therapy is an effective treatment for patients with intermittent claudication. Neuromuscular electrical stimulation as an adjunct to local standard care may benefit patients with mild intermittent claudication.

## Introduction

Peripheral arterial disease (PAD) is a common condition caused predominantly by atherosclerotic arterial stenosis or occlusion, with resultant reduction in blood flow to the affected limb^1^. It presents a significant global health burden, affecting over 200 million people worldwide^2^. Risk factors include smoking, dyslipidaemia, hypertension and diabetes. These individuals are at higher risk of other cardiovascular events^3^.

Intermittent claudication (IC) is the commonest symptom of PAD, affecting 5–10 per cent of people over 50 years of age^4^, whereby patients experience exertional muscular leg pain relieved by rest. Around 5–10 per cent go on to develop chronic limb-threatening ischaemia, characterized by ischaemic rest pain and/or tissue loss^5^. In some cases, this may result in limb amputation with associated impact on quality of life (QoL) and mortality^4^. PAD is the single largest cause of limb amputation, with diabetic patients at highest risk^2^.

The UK National Institute for Health and Care Excellence (NICE)^6^, the European Society for Vascular Surgery (ESVS)^7^ and the US Society for Vascular Surgery (SVS)^8^ guidelines all recommend BMT (best medical therapy; medication) and stress the benefits of supervised exercise therapy (SET). Despite the evidence, SET is not universally available and patient uptake is relatively poor^9,10^. Hence ‘real-world’ standard care is often BMT only^9,11,12^, despite SET being a highly cost-effective treatment^13^.

Neuromuscular electrical stimulation (NMES) devices are an emerging technology that may benefit patients with IC by increasing the distance walked before symptomatic limitation and thus improving QoL^14^. While evidence is limited for NMES as a treatment for IC, previous studies suggest improvements in the total distance walked before stopping due to IC (absolute walking distance (AWD)), the distance walked before the onset of IC (initial claudication distance (ICD)) and QoL after using an NMES device^14–16^.

Technological advances have allowed the development of portable, inexpensive and safe NMES units suitable for domiciliary use^17^. The aim of the Neuromuscular Electrical Stimulation for Intermittent Claudication (NESIC) trial is to assess the clinical efficacy of an NMES device as an adjunct to the local standard care available at study sites, in improving walking distance in patients with IC.

## Methods

### Design

The trial design has been published previously^18^ (*Supplement S1*). The NESIC trial was a multicentre, RCT in 11 hospitals in the UK (*eTable S1*). Participants were randomized 1:1 to either local standard care or local standard care and NMES. The trial was designed and overseen by a trial management group, an independent trial steering committee and an independent data monitoring committee (*eAppendix S2*). The study followed the CONSORT reporting guideline.

### Participants

Patients with IC attending vascular surgery outpatient clinics were screened. Patients aged 18 years or older were eligible if they had a positive Edinburgh Claudication Questionnaire, and an ankle–brachial pressure index (ABPI) of <0.9 or positive stress test (fall in ankle pressure greater than 30 mmHg, 40 seconds post 1-minute treadmill at 10 per cent gradient, 4 km/h). Exclusion criteria included pregnancy, inability to complete the treadmill test or SET, severe IC requiring surgery, critical limb ischaemia as defined by the European Consensus Document^7^, any implanted electrical or defibrillator device, or recent lower limb injury. Patients able to walk for longer than 15 minutes at baseline were excluded (*eAppendix S3*—full inclusion/exclusion criteria). All participants provided written informed consent.

### Randomization

Participants were randomly allocated 1:1, using random block sizes and stratified by recruitment site. Those sites with SET (*n* = 6) continued to provide this intervention as per their standard care. All patients received BMT as per local guidelines (*eTable S2*). The randomization took place via the Inform system (the electronic case report form database for the study), which was programmed using a randomization list prepared by an independent statistician and neither the research team nor the trial statistician or the patients were aware of the allocation sequence ahead of the allocation.

## Interventions

### Best medical therapy

All participants received BMT including exercise advice, smoking cessation, statin, antiplatelet and management of hypertension and diabetes mellitus, according to local standard care.

### Supervised exercise therapy

Participants at SET centres were enrolled into the local SET programme; the number of weekly sessions and duration varied between centres (*eTable S3*). Sessions typically involved a minimum 30-minute circuit of lower limb exercises led by a physiotherapist or allied healthcare worker.

### Neuromuscular electrical stimulation

Participants randomly assigned to the NMES group were given the Revitive^TM^ IX device (Actegy, Bracknell, UK), a class II, CE-certified medical device. It delivers a 30-minute pre-programmed NMES session to lower limb muscles through direct skin contact footpads in seated participants. The intensity of impulses (0–99) is user controlled. Therapeutic benefit is deemed when impulses are sufficient to cause calf muscle contraction. The IsoRocker feature allows the device to tilt back and forth as the muscles contract and relax.

The device was to be used for at least one 30-minute session daily (up to a maximum of six sessions daily) for 3 months (treatment period); diabetic patients were encouraged to use NMES for a minimum of two 30-minute sessions daily.

Participants in both groups were followed up at 3 months (end of treatment period), 6 and 12 months post-randomization (end of study participation).

### Follow up

Assessments at these time points included the standardized treadmill test, ABPI, peripheral pulse examination, quality-of-life questionnaires, haemodynamic assessments, review of patient diaries, assessment of adverse events and concomitant medications.

The 12-month follow-up appointment marked the end of study participation (*eAppendix S4*).

### Outcomes

The primary outcome measure was AWD at 3 months using the standardized Gardner–Skinner treadmill test; beginning at 3.2 km/h at a 0 per cent incline with the incline increasing by 2 per cent every 2 minutes, for a total of 15 minutes. Patients indicated when they first experienced claudication pain (ICD) and the test would finish when this prevented continuation (AWD). To prevent bias, patients were blinded to the AWD.

Secondary outcomes included ICD, compliance to interventions, QoL and haemodynamic changes. ICD was assessed by the Gardner–Skinner treadmill test at randomization, 3, 6 and 12 months. Device compliance during the treatment period was assessed by self-report patient diaries, cross-checked with voltage/current data loggers. Patients were able to continue to use the device following the 3-month treatment period.

The generic EuroQoL Five Dimensions Five-Level (EQ-5D-5L), the Medical Outcomes Study 36-Item Short-Form (SF-36) Health Survey and the disease e-specific Intermittent Claudication Questionnaire (ICQ) (*eTable S4*, *eAppendix S5 and eAppendix S6*) were collected at each follow up.

Due to the COVID-19 pandemic, study sites replaced on-site visits with telephone calls (*eAppendix S7*).

A health economic analysis was prespecified in the trial protocol, but the results are not reported in the current article.

### Statistical analysis

The sample size was estimated assuming the mean AWD in the control group would be 200 m at 3 months^19^, with a standard deviation of 120 m^20^. Anticipating a 10 per cent loss to follow-up, we estimated 192 participants would be required to have 90 per cent power with a two-sided alpha level of 5 per cent to detect a difference of 60 m in mean AWD at 3 months between intervention and control group, with a common standard deviation of 120 m.

The AWDs of participants who walked more than 15 minutes on the treadmill at 3 months were censored at 790 m. We hypothesized that there would be an improvement in AWD at 3 months in the treatment group compared to the control group in patients with IC by using a prespecified Tobit regression model to incorporate the right-censored data. The model included the AWD baseline measurement, a treatment indicator and the type of centre (SET *versus* non-SET) as covariates. As the data collected for AWD showed a right-skewed distribution, a square root transformation was used to normalize the data and used for the regression Tobit model and multilevel Tobit model.

A predefined statistical analysis plan (SAP) was written. Any analysis not in the SAP is defined as post hoc.

The secondary outcome of ICD was analysed using a multilevel Tobit regression at 3, 6 and 12 months to incorporate the right-censored distribution of data. For ABPI, mixed models were used. As the ABPI data showed a skewed distribution, log transformation was used for the analyses.

Mixed models for each of the quality-of-life scores were performed to investigate changes in QoL over time, treating patient and centre as random effects, and QoL scores at baseline, time, treatment and interaction of treatment and time as fixed effects.

Subgroup analysis to investigate the effect of the intervention among NMES + SET + BMT, NMES + BMT, SET + BMT and BMT was performed. Seven subgroup analyses were performed in the intention-to-treat (ITT) population for the primary outcome (AWD), measured at 3 months using Tobit regression models, five were originally described in the SAP and two were added later as post-hoc analyses.

A post-hoc analysis was performed with baseline AWD divided into short, medium and long distances (<25 per cent, 25–75 per cent and >75 per cent, respectively). For each stratum a Tobit regression for the transformed right-censored AWD at 3 months was performed.

All analyses were performed on an ITT basis with STATA software, version 17 (StataCorp), with statistical significance set at a two-sided alpha level of 5 per cent.

## Results

### Screening

**Fig. 1:**
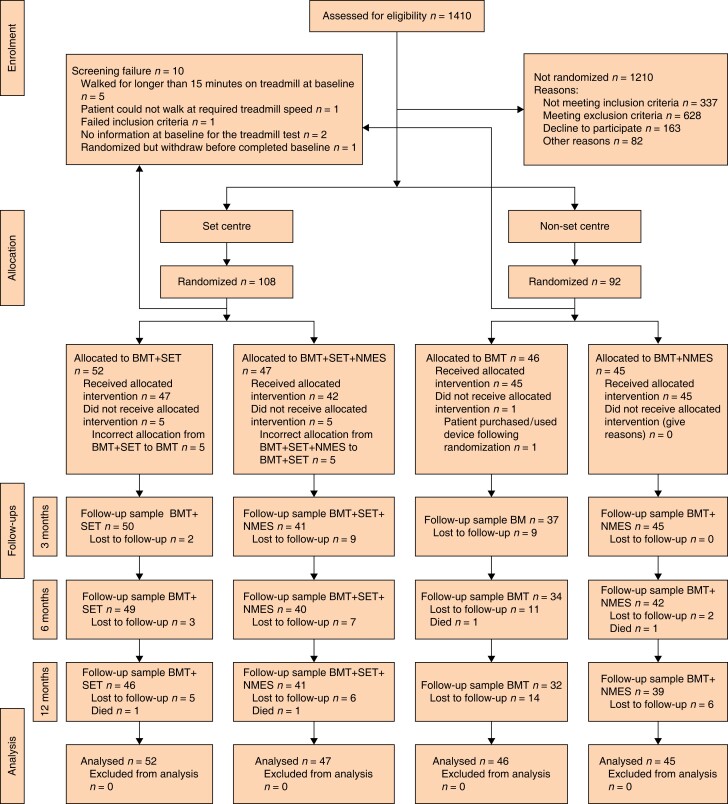
Cumulative numbers of patients who had been lost to follow-up and had died by each follow-up time point Ten patients were excluded post-randomization. Patients at SET centres attended their first SET class within 2 weeks from randomization. Treatment protocol violations occurred in 6 patients in the control group (SET and non-SET groups) and in 5 patients in the SET intervention group.

From 2 February 2018 until 31 March 2020, a total of 1410 patients were screened, and 200 consented and were subsequently randomized into the trial at 11 participating centres. However, 10 patients were removed from the analysis after randomization as they were identified as screening failures (*Fig. 1*). Of the 190 patients randomized, 160 patients had analysable primary outcome data (both baseline and 3-month treadmill test data). The ITT analysis was carried out using the data of these 160 participants. The most common reasons for exclusion were an ABPI score of 0.9 or higher (326 patients), a co-morbid disease prohibiting treadmill assessment and/or attending SET classes (166 patients), declining to participate or research team could not contact (mainly due to travel and/or time commitments of attending SET; 163 patients) and severe IC requiring invasive intervention (156 patients).

Baseline characteristics were similar in both groups (*Table 1* and *eTable S5*). The majority were former smokers and had a medical history of hypertension and dyslipidaemia. A supplementary table (*eTable S5b*) presents the baseline characteristics for those analysed. The final 12-month patient follow-up examination was completed on 31 March 2021.

**Table 1:** Baseline characteristics of trial participants

Characteristic	Treatment: NMES + BMT and NMES + BMT + SET *n* = 92	Control: BMT and BMT + SET *n* = 98
Age (mean ± s.d.)	68.17 ± 8.84 years	67.44 ± 9.44 years
BMI (mean ± s.d.)	28.10 ± 5.12 kg/m^2^	28.63 ± 6.66 kg/m^2^
**Sex**		
Female	22 (23.9%)	28 (28.6%)
Male	70 (76.1%)	70 (71.4%)
**Smoking status**		
Current	22 (23.9%)	34 (34.7%)
Former	64 (69.6%)	58 (59.2%)
Never	6 (6.5%)	6 (6.1%)
ABPI* (mean ± SD)		
Right	0.72 ± 0.18	0.76 ± 0.21
Left	0.76 ± 0.21	0.77 ± 0.22

ABPI, ankle–brachial pressure index; BMT, best medical therapy; NMES, neuromuscular electrical stimulation; SET, supervised exercise therapy. Percentages may not total to 100 because of rounding. *Information on ABPI was missing for 2 patients in the treatment group (left and right ABPI) and 2 and 1 patient(s) in the control group (right and left ABPI), respectively.

### Primary outcome

The Tobit regression model indicated that there was no statistically significant difference in the AWD at 3 months between the two study groups (NMES + SET + BMT and NMES + BMT *versus* SET + BMT and BMT). Patients in the treatment group (device) had improved AWD at 3 months compared to those in the control group (no device) (0.83 units; 95 per cent c.i., −0.67 to 2.34; *P* = 0.28; *Table 2*). This finding was not statistically significant at a significance level of 5 per cent. When considering the repeated measures in time, and further adjustment for age, gender, BMI, smoking status and treatment by time interaction and AWD at baseline with the use of a multilevel Tobit model, there were no changes to the results.

**Table 2:** **Output of the right censored**† Tobit regression model for AWD* **at 3 months for the ITT population (*n* = 160)**

	Tobit regression (AWD square root transformation)
	Model 1
Square root of AWD at baseline	0.78 [0.65,0.92] *P* < 0.001
**Treatment**	
Control^‡^: BMT and BMT + SET	
Treatment: NMES + BMT and NMES + BMT + SET	0.83 [−0.67,2.34] *P* = 0.28
**Type of centre**	
Non-SET	
SET	3.29 [1.77,4.82] *P* < 0.001
Constant	4.05 [1.62,6.48] *P* < 0.001

AWD: absolute walking distance; ITT: intention-to-treat; SET: supervised exercise therapy. Tobit regression model: square root of AWD at 3 months = intercept + square root of AWD (baseline) + Treatment + Type of centre. *The square root transformation of AWD was used for baseline and 3 months measurements. Square root transformation variables satisfy the assumptions of the Tobit model. ^†^Right censoring set up at (28.106939) square root of AWD at 790 m. ^‡^Control: local available best medical therapy (BMT and BMT + SET) as reference category. ^§^Non-SET exercise centres as reference category.

However, there was a significant increase in the AWD at 3 months (square root; 3.29 units; 95 per cent c.i., 1.77 to 4.82; *P* < 0.001) for patients recruited at SET centres compared with patients recruited at non-SET centres (*Table 2*).

### Secondary outcomes

When considering the repeated measures of ICD in time, and adjusting for age, gender, BMI, smoking status, treatment by time interaction and ICD at baseline using a multilevel Tobit model for ICD at 3, 6, and 12 months, we found that there was no statistically significant difference between treatment and control groups (*eTable S6*).

Participants’ right ABPI (log-transformed for normality) significantly increased over the follow-up period, irrespective of treatment group, by 0.07 (95 per cent c.i., 0.02 to 0.12; *P* = 0.01) at 6 months and by 0.07 (95 per cent c.i., 0.02 to 0.13; *P* = 0.01) at 12 months (*eTable S7*). However, there were no statistically significant findings between the treatment group compared to the control group, or any significant findings for left ABPI (*eTable S8*).

Quality-of-life outcomes are summarized in *Table 3* and *eTable S9* (SF-36 domain scores). There was no statistically significant difference in EQ-5D-5L or SF-36 scores between the treatment groups over the follow-up period (*Table 3*), although there was a statistically significant difference in the EQ-5D-5L health scale following the 3-month treatment period indicating a better health score in the treatment group compared with the control group (7.1; 95 per cent c.i., 1.8 to 12.4; *P* = 0.01), but this was not sustained at 6 or 12 months. Disease-specific ICQ score decreased in both groups, indicating less pain from baseline throughout the follow-up period. *Table 3* shows that there was a statistically significant difference in ICQ score at 12 months between the treatment and the control groups (4.3; 95 per cent c.i., 0.7 to 7.9; *P* = 0.02).

**Table 3:** Summary of disease-specific and generic patient-reported quality of life outcomes*

Outcome	Treatment: NMES + BMT and NMES + BMT + SET		Control: BMT and BMT + SET		Between-group difference in score (95% c.i.)†
	No. of patients	Score	No. of patients	Score	
**ICQ health scale‡**					
Baseline	90	41.98 ± 13.26	94	45.92 ± 13.09	
3-months	84	36.55 ± 13.86	82	41.33 ± 14.52	−1 (−4.5 to 2.4) *P* = 0.56
6-months	78	35.20 ± 15.07	77	39.27 ± 14.51	−0.1 (−3.7 to 3.4) *P* = 0.94
12-months	76	36.99 ± 17.38	76	36.21 ± 16.45	4.3 (0.7 to 7.9) *P* = **0.02**
**EQ-5D-5L health scale§**					
Baseline	91	69.73 ± 18.03	97	69.61 ± 17.69	
3 months	85	74.02 ± 15.13	84	66.11 ± 21.09	7.1 (1.8 to 12.4) *P* = **0.01**
6 months	79	73.13 ± 19.32	77	68.36 ± 20.85	3.5 (−1.9 to 8.9) *P* = 0.21
12 months	77	70.40 ± 20.98	76	68.03 ± 19.61	1.9 (−3.5 to 7.4) *P* = 0.49
**EQ-5D-5L health index¶**					
Baseline	91	0.63 ± 0.20	97	0.62 ± 0.20	
3 months	85	0.66 ± 0.20	84	0.62 ± 0.21	0.04 (−0.02 to 0.09) *P* = 0.17
6 months	79	0.65 ± 0.22	78	0.66 ± 0.18	−0.02 (−0.07 to 0.04) *P* = 0.56
12 months	77	0.65 ± 0.26	76	0.66 ± 0.20	0.002 (−0.05 to 0.05) *P* = 0.94
**SF-36 Physical Component Summary‖**					
Baseline	91	35.71 ± 8.22	95	36.14 ± 7.90	
3 months	84	38.80 ± 8.87	84	37.42 ± 8.48	1.7 (−0.6 to 4) *P* = 0.14
6 months	79	39.47 ± 9.74	77	37.62 ± 9.85	2.3 (0.02 to 4.7) *P* = 0.048
12 months	76	38.16 ± 9.98	75	39.46 ± 9.40	−0.6 (−3 to 1.7) *P* = 0.6
**SF-36 Mental Component Summary‖**					
Baseline	91	52.06 ± 11.61	95	49.75 ± 12.47	
3 months	84	52.99 ± 10.05	84	48.24 ± 13.15	2.1 (−0.9 to 5.1) *P* = 0.18
6 months	79	52.79 ± 10.73	77	49.09 ± 10.90	1.3 (−1.8 to 4.3) *P* = 0.43
12 months	76	52.62 ± 11.68	75	48.90 ± 12.24	1.5 (−1.6 to 4.6) *P* = 0.34

BMT: best medical therapy; ICQ: intermittent claudication questionnaire; EQ-5D-5L: EuroQol Group 5-Dimension 5-Level questionnaire; NMES: neuromuscular electrical stimulation; SET: supervised exercise therapy; SF-36: medical outcomes study 36-item short-form health survey. *Plus–minus values are means ± SD. †The between-group differences were estimated by a mixed model adjusted for each baseline quality of life score, time, treatment and the interaction term of time and treatment as fixed effects and centre and patients as random effects. The control group was the reference group. The widths of the confidence intervals were not adjusted for multiple comparisons and should not be used for formal reference. ‡Scores on the ICQ range from 0 to 100, with higher scores indicating worse health related to intermittent claudication. §Scores on the EQ-5D-5L health scale (a visual analogue scale) range from 0 to 100, with higher scores indicating better health. ¶Scores on the EQ-5D-5L health index range from 0 to 1, with higher scores indicating better health. The EQ-5D-5L health index was calculated with the value set for England^21^. ‖Scores on the SF-36 Physical Component Summary and Mental Component Summary range from 1 to 100, with higher scores indicating better quality of life. Bold values are statistically significant results.

Serious adverse events (SAEs; *n* = 29) were reported in 24 participants, with all events being classified as either not related or unlikely to be related to the study device. The number of SAEs in the treatment group was 13 and 16 in the control arm. Most of the events required hospitalization and there were four deaths. *eTable S10* includes SAEs for the overall population categorized by treatment.

Subgroup analyses are summarized in *Table 4*. SET had a statistically significant greater impact on the AWD (after square root transformation) than NMES (−2.42 units; 95 per cent c.i., −4.32 to −0.51; *P* = 0.01). However, when NMES was used as an adjunct to BMT and SET, there was a trend towards improved walking distances in the treatment group (device), but this was not statistically significant (1.72 units; 95 per cent c.i., −0.56 to 4.01; *P* = 0.14).

**Table 4:** **Output of right-censored**† **Tobit regression model**‡ **for AWD at 3 months to assess the effects of each subgroup for the ITT population**

Independent variables	Tobit regression (square root transformation of the AWD)	
	Coeff [95% c.i.]	*P*
Square root of AWD at baseline	0.79 [0.65,0.93]	***P* < 0.001**
**Sugroup1**		
** **Non-SET*	–	–
** **SET	2.36 [0.21,4.51]	***P* = 0.03**
**Treatment**		
** **Control: BMT and BMT + SET*		
** **Treatment: NMES + BMT and NMES + BMT + SET	−0.19 [−2.45,2.06]	*P* = 0.87
**Treatment × Subgroup1^§^**		
** **Control **×** non-SET*		
** **Treatment **×** SET	1.85 [−1.18,4.88]	*P* = 0.23
**constant**	4.57 [2.00,7.13]	***P* < 0.001**
Square root of AWD at baseline	0.87 [0.66,1.07]	***P* < 0.001**
**Sugroup2^#^**		
** **BMT + SET*	–	–
** **BMT + SET + NMES	1.72 [−0.56,4.01]	*P* = 0.14
Constant	5.88 [2.67,9.08]	
Square root of AWD at baseline	0.7 [0.52,0.88]	***P* < 0.001**
Sugroup3^**^		
** **BMT*	–	–
** **BMT + NMES	−0.09 [−2.01,1.83]	*P* = 0.93
Constant	5.85 [2.92,8.78]	***P* < 0.001**
Square root of AWD at baseline	0.69 [0.51,0.87]	***P* < 0.001**
**Sugroup4^††^**		
** **BMT + SET*	–	–
** **BMT + NMES	−2.42 [−4.32,−0.51]	***P* = 0.01**
Constant	8.25 [5.45,11.06]	***P* < 0.001**
Square root of AWD at baseline	0.86 [0.68,1.03]	***P* < 0.001**
**Sugroup5^‡‡^**		
** **BMT + NMES*	–	–
** **BMT + SET + NMES	4.25 [2.23,6.27]	***P* < 0.001**
Constant	3.35 [0.39,6.31]	***P* = 0.03**
Square root of AWD at baseline	0.9 [0.68,1.11]	***P* < 0.001**
**Sugroup6^§§^**		
** **BMT*	–	–
** **BMT + SET + NMES	−4.1 [−6.56,−1.64]	***P* < 0.001**
Constant	7.16 [3.79,10.53]	***P* < 0.001**
Square root of AWD at baseline	0.69 [0.47,0.91]	***P* < 0.001**
**Sugroup7^##^**		
** **BMT*	–	–
** **BMT + SET	2.34 [0.05,4.63]	***P* = 0.04**
Constant	5.94 [2.32,9.56]	***P* < 0.001**

AWD: absolute walking distance; BMT: best medical therapy; ITT: intention-to-treat; NMES: neuromuscular electrical stimulation; SET: supervised exercise therapy. *Indicates the reference category. †Right censoring set up at (28.106939) square root of AWD at 790 m. ‡Tobit regression model: square root of AWD at 3 months = intercept + square root of AWD (baseline) + Subgroup + residual. §Subgroup 1: Non-SET *versus* SET; Non-SET as reference category; 148 uncensored observations; 12 right-censored observations. #Subgroup 2: BMT + SET *versus* BMT + SET + NMES; BMT + SET as reference category; 79 uncensored observations; 11 right-censored observations. **Subgroup 3: BMT *versus* BMT + NMES; BMT as reference category; 69 uncensored observations; 1 right-censored observation. ††Subgroup 4: BMT + SET *versus* BMT + NMES; BMT + SET as reference category; 85 uncensored observations; 3 right-censored observations. ‡‡Subgroup 5: BMT + NMES *versus* BMT + SET + NMES; BMT + NMES as reference category; 72 uncensored observations; 8 right-censored observations. §§Subgroup 6: BMT + SET + NMES *versus* BMT; BMT + SET + NMES as reference category; 63 uncensored observations; 9 right-censored observations. ##Subgroup 7: BMT *versus* BMT + SET; BMT as reference category; 76 uncensored observations; 4 right-censored observations. Bold values are statistically significant results.

Compliance was measured for all interventions. Participants at SET centres were deemed compliant if they attended 50 per cent or more sessions, and if participants completed at least 75 per cent of their recommended level of NMES usage. Of 99 participants that attended SET, 69 were compliant (69.7 per cent) with data missing for 11 patients (11.1 per cent). Of 92 participants using the device, 68 were compliant (73.9 per cent), with data missing for 12 patients (13.0 per cent). Patients reported good tolerability to device use; 87.5 per cent stated it was ‘very easy’ to use as reported in the device experience questionnaire.

### Post-hoc analysis

A post-hoc analysis was performed looking at stratification of baseline AWD. The AWD at baseline was divided into three strata: short, medium and long distances (set at <25 per cent, 25–75 per cent and >75 per cent, respectively) using the descriptive statistics in *eTables S11*, *S12*.

For patients that could not walk further than 100 m at baseline, there was no clear statistical difference between the two treatment arms, or between type of centre (SET *versus* non-SET) (*eTable S13*). For patients with a medium baseline AWD, there was no statistically significant difference in AWD at 3 months between the two treatment arms, but there was a statistically significant difference between type of centre (*eTable S14*). There were statistically significant differences between both treatment arms and type of centre for those patients able to walk a longer baseline AWD (*eTable S15*).

## Discussion

### Principal findings

This trial showed that SET is an effective treatment for patients with IC. The addition of NMES may have an adjuvant benefit on AWD, particularly in patients with mild IC. From the subgroup analysis we can conclude that SET has a greater impact in the improvement of AWD both alone or in combination with NMES. Exercise advice alone has the lowest impact on the improvement of AWD.

### Strengths and weaknesses of the study

Our trial has several limitations. First, the AWD that was used as the primary outcome measure showed a large range in both groups at baseline, with a right-skewed distribution. We did not stratify by baseline AWD for the primary outcome analysis. Second, only 160 participants had analysable primary outcome data due to missing treadmill data at baseline and/or 3 months. The number of participants lost to follow-up was higher than first anticipated. Additionally, the COVID-19 pandemic resulted in local centre policy, at some participating centres, dictating that participants were not permitted to attend study appointments face to face. Instead, remote visits were performed with physical assessments, such as the treadmill test, being missed. Certain secondary outcomes, such as haemodynamic measures, were not adjusted for centre effects and thus may not account for centre–centre variability. Finally, there was the absence of a sham device comparator. This was considered during the protocol design but was deemed impractical to implement due to the patient setting the stimulation level to a threshold where calf contractions are visible (the IsoRocker feature allows the device to tilt back and forth).

The main strength of the NESIC trial is that this is the first moderately sized RCT looking at the adjuvant benefit of NMES in patients with IC. The results of this trial are generalizable across vascular units that provide SET and those that provide BMT only. A further strength is that compliance data were collected separately for NMES, SET and exercise advice, with clear definitions on what is deemed as compliant.

### Comparisons with other studies

The primary analysis suggests NMES has no additional benefit overall in individuals with IC receiving local standard care. This finding is divergent from the RCT by Babber *et al.*^16^, which found a significant improvement in walking distances after using the device for 30 min daily for 6 weeks, when used both independently and also as an adjunct to SET. Considering possible reasons for this discrepancy, it is noted that the previous study did not reach the target sample size due to the limited recruitment period, while in this study we have hypothesized that there may be reduced compliance with exercise advice when supplied with an NMES device. Similarly, quality-of-life findings were mixed and did not show a strong sign of benefit as in Babber *et al*. study^16^. An RCT performed by Cheetham *et al*.^19^, however, showed significant disease-specific quality-of-life improvements in participants receiving SET compared to exercise advice alone.

Compliance with intervention is an important consideration when managing patients with PAD. In this trial, 69.7 per cent of patients with access to SET met the definition of compliance (50 per cent or more classes held by the site). Current data on patient adherence to SET programmes is problematic due to differences in defining compliance among studies and the large variation in SET programme duration. Harwood *et al*.^22^ conducted a systematic review of 67 studies in 2016 that found an average of 75.1 per cent patients reportedly completing the SET programme, although only one article defined a minimal attendance required for completion.

Compliance with NMES in this study was 73.9 per cent, which was less than what was observed in the 6-week pilot study (97 per cent) and subsequent RCT (96 per cent)^16^. Throughout the duration of the trial, no participants contacted the local research team to seek additional support. The majority of device users (87.5 per cent) agreed that the device was ‘very easy’ to use and 63.6 per cent stated that they could have used the device more frequently. Compliance to exercise advice was the lowest of the three treatments (52.1 per cent), but there was a high percentage of missing data from the patients’ self-reported diaries (20.5 per cent).

A sensitivity analysis, using only the compliance rules for SET and NMES, with all patients receiving exercise advice, showed no clear statistical differences to the main analysis (including all seven subgroup analyses).

### Meaning of the study

The results of our study add to the growing body of evidence that SET has a significant benefit on walking distances of patients with IC^9–11,23^. However, many patients with IC do not have access to SET, mainly due to lack of local provision^9,10^, and therefore the development of novel technologies such as NMES, which can be delivered at the level of the individual patient, to be used as an adjunct to local available therapy may have a role in the first-line treatment of IC.

In the current trial we found that there was no overall significant improvement in walking distances in those patients using NMES as an adjunctive treatment to SET. No statistically significant effect was observed, but there was a trend suggesting a potential advantage to combined treatment. Interestingly, our post-hoc analysis suggests the response to SET and NMES appears to be dependent on baseline walking ability, and that these treatment options may be better for patients with IC able to walk longer distances. This, taken with the previous body of evidence of improved walking distances^15,16^, suggests this may be an area for further investigation.

In conclusion, this multicentre, randomized trial demonstrates the clear benefit of SET for patients with IC. NMES appears to be beneficial both as an adjunct to SET and on its own in patients with longer baseline walking distances. This is of particular importance for patients with mild IC and in vulnerable groups that may feel unable to travel or feel uncomfortable attending SET and/or commercial gyms in light of the COVID-19 pandemic, as NMES devices are widely available and can be used in a home setting.

### Unanswered questions and future research

Further studies are needed to confirm the effectiveness of NMES in combination with SET, and in patients with IC who have good baseline walking distances in a larger sample size.

## Supplementary Material

znad299_Supplementary_Data

## Data Availability

All data requests should be submitted to the corresponding author for consideration. Access to anonymized data may be granted following review. Data-access requests are handled on a case-by-case basis and will be reviewed by the corresponding author, Trial Management Group and sponsor (Imperial College London). A record of all access to data will be maintained by the Imperial College Archive team.
