# Supplementary material for: Neuromuscular Electrical Stimulation for Intermittent Claudication (NESIC): multicentre, randomized controlled trial
Source: Br J Surg. 2023 Sep 25;110(12):1785–92. doi: 10.1093/bjs/znad299 (PMC10638544; doi:10.1093/bjs/znad299)
Supplement: znad299_Supplementary_Data [file znad299_supplementary_data.docx]

**Neuromuscular electrical stimulation as an adjunct to best available treatment in improving walking distances in patients with intermittent claudication (NESIC): a multicentre, randomised controlled trial**

Laura Burgess MSc^1,2^, Adarsh Babber PhD^1,2^, Joseph Shalhoub PhD^1,2^, Sasha Smith BSc^1,2^, Consuelo Nohpal de la Rosa MSc^3^, Francesca Fiorentino PhD^1,3,4^, Bruce Braithwaite MChir^5^, Ian Chetter MD^6^, James Coulston MSc^7^, Manjit Gohel MD^8^, Robert Hinchliffe MD^9^, Gerard Stansby MChir^10^, Alun H Davies DSc^1,2^; for the NESIC Trial Investigators*

^1^Department of Surgery and Cancer, Imperial College London, London, UK

^2^Imperial Vascular Unit, Imperial College Healthcare NHS Trust, London, UK

^3^Imperial Clinical Trials Unit, Imperial College London, London, UK

^4^Nightingale-Saunders Clinical Trials & Epidemiology Unit (King’s Clinical Trials Unit), King’s College London, London, UK

^5^Nottingham University Hospitals NHS Trust, Nottingham, UK

^6^Hull York Medical School, University of Hull / Hull University Teaching Hospital NHS Trust, Hull, UK

^7^Somerset NHS Foundation Trust, Taunton, UK

^8^Cambridge University Hospitals NHS Foundation Trust, & NIHR Cambridge Biomedical Research Centre, Cambridge, UK

^9^North Bristol NHS Trust, Bristol, UK

^10^The Newcastle Upon Tyne Hospitals NHS Foundation Trust, Newcastle, UK

*A complete list of NESIC Trial Investigators is provided in eAppendix 1.

**Corresponding author.** Professor Alun Davies, Section of Vascular Surgery, Department of Surgery and Cancer, Imperial College London, Charing Cross Hospital, London, W6 8RF, UK; Tel: +44 (0) 208 3311 7320; Fax: +44 (0)208 3311 7362; a.h.davies@imperial.ac.uk **ORCID ID**; 0000-0001-5261-6913

This supplementary material has been provided by the authors to give readers additional information about their work.

**Supplementary Materials - Index**

| **Supplementary Methods** |  |
| --- | --- |
| Supplement 1. NESIC protocol | *pag. 3-74* |
| **Supplementary Appendixes** |  |
| eAppendix 1. List of investigators | *pag. 75* |
| eAppendix 2. Trial committees | *pag. 75* |
| eAppendix 3. List of inclusion and exclusion criteria | *pag. 76* |
| eAppendix 4. End of Study | *pag. 76* |
| eAppendix 5. EuroQol-5D-5L | *pag. 77-79* |
| eAppendix 6. Intermittent Claudication Questionnaire (ICQ) | *pag. 80-81* |
| eAppendix 7. Impact of COVID-19 | *pag. 82* |
| eAppendix 8. Patient and public involvement (PPI) | *pag. 82* |
| eAppendix 9. CONSORT checklist | *pag. 83-85* |
| **Supplementary Figures and Tables** |  |
| eTable 1. NESIC study: recruiting centres | *pag. 86* |
| eTable 2. Summary of treatment allocation | *pag. 87* |
| eTable 3. Supervised Exercise Therapy (SET) localised programs | *pag. 88* |
| eTable 4. Summary of quality of life tools using in NESIC study | *pag. 89* |
| eTable 5. Additional baseline characteristics of the trial participants* | *pag. 90* |
| eTable 5b. Additional baseline characteristics of those who were analysed* | *pag. 91* |
| eTable 6. Output of the right censored^1^ multilevel Tobit model to assess the effects of baseline characteristics for ICD** at 3, 6, and 12 months for the ITT population (N=159) | *pag. 92* |
| eTable 7. Output of linear mixed model^1^ for changes in the transformed log Right Ankle Brachial Pressure Index (ABPI)* between baseline and follow up periods (3, 6 and 12 months) for the ITT population N=159 | *pag. 93* |
| eTable 8. Output of linear mixed model^1^ for changes in the transformed log Left Ankle Brachial Pressure Index (ABPI)* between baseline and follow up periods (3, 6 and 12 months) for the ITT population N=160 | *pag. 94* |
| eTable 9. Summary of Quality-of-life outcomes – sub-domains of SF-36 | *pag. 95-96* |
| eTable 10. Serious adverse events – overall population by as treated | *pag. 97* |
| eTable 11. Descriptive statistics of Absolute Walking Distance (AWD) at baseline | *pag. 98* |
| eTable 12. Stratification of Absolute Walking Distance (AWD) by low, medium, and high distance | *pag. 99* |
| eTable 13. Output of the right censored^1^ Tobit regression model^2^ for Absolute Walking Distance (AWD)** at 3 months for patients who walked a short distance at baseline in the ITT population (N=40) | *pag. 100* |
| eTable 14. Output of the right censored^1^ Tobit regression model^2^ for Absolute Walking Distance (AWD)** at 3 months for patients who walked a medium distance at baseline in the ITT population (N=80) | *pag. 101* |
| eTable 15. Output of the right censored1 Tobit regression model^2^ for Absolute Walking Distance (AWD)** at 3 months for patients who walked a long distance at baseline in the ITT population (N=40) | *pag. 102* |
| **References** |  |
| eReferences | *pag. 103* |

**Supplementary Methods**

Supplement 1. NESIC protocol

CLINICAL STUDY PROTOCOL


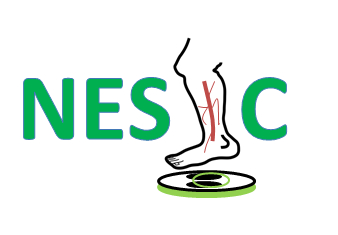


**Full Study Title:** A Multicenter Randomised Controlled Study: Does **N**euromuscular **E**lectrical **S**timulation Improve the Absolute Walking Distance in Patients with **I**ntermittent **C**laudication (NESIC) compared to best available treatment?

**Short Study title / Acronym:** NESIC

**Product:** Revitive IX

**Sponsor:** Imperial College London

**Version no:** 6.0

**Protocol Date:**  24 MAR 2020

**Property of Imperial Clinical Trials Unit (ICTU)**

**May not be used, divulged or published without the consent of ICTU**

**This protocol has regard for the HRA guidance**

This project is funded by the Efficacy and Mechanism Evaluation (EME) Programme, an MRC and NIHR partnership. The views expressed in this publication are those of the author(s) and not necessarily those of the MRC, NHS, NIHR or the Department of Health.

# Research Reference numbers

| **IRAS ID:** | **229435** |
| --- | --- |
| **REC Reference number:** | **17/LO/1918** |
| **ISRCTN number** | **18242823** |
| **Clinical trials.gov number** | **NCT03446027** |
| **Sponsor Protocol number:** | **17HH4216** |
| **Funder reference:** | **EME 15/180/68** |

# Revision summary

| **Protocol Version** | **Date** | **Revision summary** |
| --- | --- | --- |
| V1.0 | 16/10/2017 | N/A – Original Protocol |
| V2.0 | 05/12/2017 | Revision of statistical analysis section and change of Bristol Trust name |
| V3.0 | 22/03/2018 | Dosage clarification for patients with diabetes. |
| V4.0 | 07/09/2018 | Add three new participating NHS organisations |
| V5.0 | 09/09/2019 | Additional exclusion criteria. SET centres permitted to recruit nonSET patients. |
| V6.0 | 24/03/2020 | To allow 3 Month, 6 Month and 12 Month visits to take place remotely (i.e. over the telephone completely or in combination with postal questionnaires) in the event that the participant is unable to attend in clinic or the site is unable to accommodate the on-site visit. |

Keywords:

Peripheral arterial disease; intermittent claudication; neuromuscular electrical stimulation; supervised exercise therapy

# CONTACT LIST

**Chief Investigator**

Professor Alun H Davies

Room 4E4, 4th Floor East Wing

Charing Cross Hospital,

Fulham Palace Road,

London W6 8RF

Tel: 020 3311 7309

Co-Investigators:

Mr Adarsh Babber

Mrs Laura Burgess

Mr Bruce Braithwaite

Dr Richard Simpson

Professor Ian Chetter

Mr James Coulston

Dr David Epstein

Dr Francesca Fiorentino

Mr Manj Gohel

Professor Robert Hinchliffe

Professor Peter Holt

Ms Sarah Horgan

Mr James Metcalfe

Ms Nandita Pal

Mr Dynesh Rittoo

Mr Joseph Shalhoub

Professor Gerard Stansby

**Sponsor**

| Joint Research Compliance Office  Imperial College London and Imperial College Healthcare NHS Trust  Room 215, Level 2, Medical School Building  Norfolk Place  London, W2 1PG  Email: [jrco@ic.ac.uk](mailto:jrco@ic.ac.uk)  Becky Ward  Research Governance Manager  Tel: 0207 594 9459  Email: [becky.ward@imperial.ac.uk](mailto:becky.ward@imperial.ac.uk) |
| --- |

**Funder**

| Efficacy and Mechanism Evaluation (EME) Programme, an MRC and NIHR partnership  National Institute for Health Research  Evaluation, Trials and Studies Coordinating Centre  University of Southampton  Alpha House, Enterprise Road  Southampton, SO16 7NS |
| --- |

**Trial Coordination / Study Manager**

| Miss Laura Burgess  Imperial College London, Academic Section of Vascular Surgery  Room 14, 4th Floor East Wing, Charing Cross Hospital  Fulham Palace Road, London, W6 8RF  Tel: 0203 311 5208  Email: l.burgess@imperial.ac.uk |
| --- |

**Senior Statistician**

| Dr Francesca Fiorentino  Statistician and Epidemiologist  Imperial College Trial Unit & Division of Surgery  Queen Elisabeth the Queen Mother Building (10th Floor/1091)  St Mary’s Hospital, Imperial College London  Praed Street, London W2 1NY  Tel: 0203 3123761 |
| --- |

**Health Economist**

| Dr David Epstein  Department of Applied Economics  Faculty of Economics and Business Sciences  University of Granada  Email: davidepstein@ugr.es |
| --- |

**Device Manufacturing Facilities**

| Actegy Limited  REFLEX, Cain Road  Bracknell  RG12 1HL  United Kingdom  Name of contact person: Roseanna Penny  Title: Director  Tel: 01344 636 940  roseanna.penny@actegy.com |
| --- |

**Other Vendors/suppliers**

| Facility name: Moor Instruments Ltd  Address: Millwey, Axminster, EX13 5HU, United Kingdom  Tel: (general) 01297 35715  Fax: (general) 01297 35716  Name of contact person: Brian Lock  Title: Senior Sales Engineer  Tel: 01297 630915  Mobile: 07740 920 832  Fax: 0129735716 |
| --- |
| Supplier name: THE 3rd DEGREE LIMITED  Address: 4 Tregarne Terrace, St Austell, Cornwall, PL25 4B  Name of contact person: Philip Martin  Title: Managing Director  Email: phil@the3rddegree.co.uk |

**Protocol development group**

| Professor Alun Davies  Ms. Francine Heatley  Ms. Rebecca Lawton  Mrs Natalia Klimowska-Nassar  Dr David Epstein  Dr. Francesca Fiorentino  Mr Adarsh Babber |
| --- |

#

# ABBREVIATIONS

| ABPI | Ankle Brachial Pressure Index |
| --- | --- |
| AE | Adverse Event |
| ADE | Adverse Device Effect |
| ASADE | Anticipated Serious Adverse Device Effect |
| AWD | Absolute Walking Distance |
| BMT | Best Medical Therapy |
| CI | Chief Investigator |
| CRF | Case Report Form |
| DMC | Data Monitoring Committee |
| DU | Duplex Ultrasound |
| EA | Exercise Advice |
| eCRF | Electronic Case Report Form |
| EQ5D-5L | EuroQoL-5D-5L |
| GMP | Good Manufacturing Practice |
| HRA | Health Research Authority |
| IC | Intermittent Claudication |
| ICD | Initial Claudication Distance |
| ICMJE | International Committee of Medical Journal Editors |
| ICTU | Imperial Clinical Trials Unit |
| ICQ | Intermittent Claudication Questionnaire |
| IPC | Intermittent Pneumatic Compression |
| LDF | Laser Doppler Flowmetry |
| NICE | National Institute for Health and Care Excellence |
| RCT | Randomised Controlled Trial |
| REC | Research Ethics Committee |
| SF-36 | Short Form 36 |
| TAMV | Time Averaged Mean Velocity |
| NMES | Neuromuscular Electrical Stimulation |
| PAD | Peripheral Arterial Disease |
| QA | Quality Assurance |
| QALY | Quality-adjusted-life-year |
| QC | Quality Control |
| SAE | Serious Adverse Event |
| SADE | Serious Adverse Device Effect |
| SAP | Statistical Analysis Plan |
| SET | Supervised Exercise Therapy |
| SmPC | Summary of Product Characteristics |
| SOP | Standard Operating Procedure |
| SSAR | Suspected Serious Adverse Reaction |
| SUSAR | Suspected Unexpected Serious Adverse Reaction |
| TMG | Trial Management Group |
| TSC | Trial Steering Committee |
| USADE | Unanticipated Serious Adverse Device Effect |

Table of Contents

[Research Reference numbers 2](#_Toc19094196)

[Revision summary 2](#_Toc19094197)

[CONTACT LIST 3](#_Toc19094198)

[ABBREVIATIONS 7](#_Toc19094199)

[TRIAL SUMMARY 11](#_Toc19094200)

[1. background 13](#_Toc19094201)

[1.1 Peripheral Arterial Disease 13](#_Toc19094202)

[1.2 Intermittent Claudication 13](#_Toc19094203)

[1.3 Treatment options for Intermittent Claudication 13](#_Toc19094204)

[1.4 Summary of current research 13](#_Toc19094205)

[1.5 Neuromuscular electrical stimulation 15](#_Toc19094206)

[1.6 Mechanistic evaluation of the device 15](#_Toc19094207)

[1.7 Technological Basis of the Product 18](#_Toc19094208)

[1.8 Clinical evaluation of NMES techniques and similar devices 18](#_Toc19094209)

[1.9 Rationale for the study 22](#_Toc19094210)

[1.10 Risk / Benefit Assessment 24](#_Toc19094211)

[2. OBJECTIVES and endpoints 24](#_Toc19094212)

[2.1 Primary Objective 24](#_Toc19094213)

[2.2 Secondary Objective 24](#_Toc19094214)

[2.3 Primary Endpoint 25](#_Toc19094215)

[2.4 Secondary Endpoints 25](#_Toc19094216)

[3. STUDY DESign 26](#_Toc19094217)

[3.1 Design 26](#_Toc19094218)

[3.2 Treatment regimens 26](#_Toc19094219)

[4. PARTICIPANT ENTRY 28](#_Toc19094220)

[4.1 Study setting and population 28](#_Toc19094221)

[5. PROCEDURES AND MEASUREMENTS 30](#_Toc19094222)

[5.1 Identification and recruitment of patients 30](#_Toc19094223)

[5.2 Screening and pre-randomisation evaluations 30](#_Toc19094224)

[5.3 Randomisation 31](#_Toc19094225)

[5.4 Other baseline investigations 32](#_Toc19094226)

[5.5 Treatment 34](#_Toc19094227)

[5.6 Follow-up 36](#_Toc19094228)

[5.7 Summary of Visit Schedule 40](#_Toc19094229)

[6. TREATMENTS 41](#_Toc19094230)

[6.1 Investigational Medical Device Details 41](#_Toc19094231)

[6.2 Labelling and Packaging 46](#_Toc19094232)

[6.3 Storage 46](#_Toc19094233)

[6.4 Permanent Discontinuation of Study Treatment and Withdrawal from Study 46](#_Toc19094234)

[7. SAFETY REPORTING 47](#_Toc19094235)

[7.1 Adverse Events 47](#_Toc19094236)

[7.2 Adverse Event Definition 47](#_Toc19094237)

[7.3 Adverse Event Recording 48](#_Toc19094238)

[7.4 Severity of Adverse Events 48](#_Toc19094239)

[7.5 Causality of Adverse Events 48](#_Toc19094240)

[7.6 Serious Adverse Event (SAE) 49](#_Toc19094241)

[7.7 Adverse Device Effect (ADE) 51](#_Toc19094242)

[7.8 Serious Adverse Device Effects (SADE) 52](#_Toc19094243)

[7.9 Anticipated Serious Adverse Device Effects (ASADE) 52](#_Toc19094244)

[7.10 Unanticipated Serious Adverse Device Effects (USADE) 53](#_Toc19094245)

[7.11 Developmental Safety Update Reports / Annual Safety Reports 53](#_Toc19094246)

[7.12 Pregnancy 53](#_Toc19094247)

[7.13 Reporting urgent safety measures 54](#_Toc19094248)

[8. STATISTICAL ANALYSES 54](#_Toc19094249)

[8.1 . Sample size and power considerations 54](#_Toc19094250)

[8.2 . Planned recruitment rate 55](#_Toc19094251)

[8.3 Statistical analysis 55](#_Toc19094252)

[9. REGULATORY, ETHICAL AND LEGAL ISSUES 58](#_Toc19094253)

[9.1 Declaration of Helsinki 58](#_Toc19094254)

[9.2 Good Clinical Practice 59](#_Toc19094255)

[9.3 Independent Ethics Committee Approval 59](#_Toc19094256)

[9.4 Regulatory Authority Approval 60](#_Toc19094257)

[9.5 HRA approval 60](#_Toc19094258)

[9.6 Non-Compliance and Serious Breaches 60](#_Toc19094259)

[9.7 Insurance and Indemnity 61](#_Toc19094260)

[9.8 Trial Registration 61](#_Toc19094261)

[9.9 Informed Consent 61](#_Toc19094262)

[9.10 Contact with General Practitioner 62](#_Toc19094263)

[9.11 Subject Confidentiality 63](#_Toc19094264)

[9.12 Data Protection and Patient Confidentiality 63](#_Toc19094265)

[9.13 End of Trial 63](#_Toc19094266)

[9.14 Study Documentation and Data Storage 63](#_Toc19094267)

[10. DATA MANAGEMENT 64](#_Toc19094268)

[10.1 Source Data 64](#_Toc19094269)

[10.2 Language 64](#_Toc19094270)

[10.3 Database 64](#_Toc19094271)

[10.4 Data Collection 65](#_Toc19094272)

[10.5 Archiving 65](#_Toc19094273)

[11. STUDY MANAGEMENT STRUCTURE 65](#_Toc19094274)

[11.1 Trial Steering Committee 65](#_Toc19094275)

[11.2 Trial Management Group 65](#_Toc19094276)

[11.3 Data Monitoring Committee 66](#_Toc19094277)

[11.4 Early Discontinuation of the Study 66](#_Toc19094278)

[11.5 Risk Assessment 66](#_Toc19094279)

[11.6 Monitoring 66](#_Toc19094280)

[11.7 Quality Control and Quality Assurance 67](#_Toc19094281)

[11.8 Peer review 67](#_Toc19094282)

[11.9 Patient and Public Involvement 67](#_Toc19094283)

[11.10 Publication and Dissemination policy 68](#_Toc19094284)

[12. REFERENCES 71](#_Toc19094285)

[SIGNATURE PAGE 1 (Chief Investigator) 74](#_Toc19094286)

[SIGNATURE PAGE 2 (SPONSOR) 75](#_Toc19094287)

[SIGNATURE PAGE 3 (STATISTICIAN) 76](#_Toc19094288)

[SIGNATURE PAGE 4 (principal INVESTIGATOR) 77](#_Toc19094289)

#

# TRIAL SUMMARY

**TITLE**: Does **N**euromuscular **E**lectrical **S**timulation Improve the Absolute Walking Distance in Patients with Intermittent **C**laudication **(NESIC)** Compared to Best Available Treatment?

**OBJECTIVE:** To assess the benefit of using a neuromuscular electrical stimulation device as an adjunct to the local standard care available at the study sites compared to local standard care alone.

**DESIGN:** This is a 1:1 multicenter randomised trial stratified by centre.

**SAMPLE SIZE:** A total of 192 participants: 96 in each arm

**INCLUSION/EXCLUSION CRITERIA**

Inclusion Criteria:

- Capacity to provide informed consent
- Aged 18 or above
- Positive Edinburgh Claudication Questionnaire
- ABPI <0.9 OR positive stress test (fall in ankle pressure >30mmHg, 40 secs post 1 min treadmill at 10% gradient, 4 km/h)

Exclusion Criteria:

- Severe IC requiring invasive intervention as determined by the treating clinician
- Critical limb Ischaemia as defined by the European Consensus Document
- Co-morbid disease prohibiting walking on a treadmill or taking part in supervised exercise therapy.
- Able to walk for longer than 15 minutes on the study treadmill assessment
- Have attended supervised exercise therapy classes in the previous 6 months
- Popliteal entrapment syndrome
- Commenced vascular symptom specific medication in previous 6 months e.g. naftidrofuryl oxalate, cilostazol
- Pregnancy
- Any implanted electronic, cardiac or defibrillator device
- Acute deep vein thrombosis
- Broken or bleeding skin including leg ulceration
- Peripheral neuropathy
- Recent lower limb injury or lower back pain
- Already using a neuromuscular electrical stimulation device

**TREATMENT/MAIN STUDY PROCEDURES**

Treatment duration: 3 months of device use

Follow-Up duration: 12 months (follow-up visits at 3, 6, and 12 months)

**SET Centre**

- Control Group 1: Exercise Advice + Supervised Exercise Therapy
- Treatment Group 1: Exercise Advice + Supervised Exercise Therapy and Neuromuscular electrical stimulation (NMES)

When recruitment is completed at SET centres, authorised SET centres will be permitted to randomise patients using a randomisation list that will allocate participants to Exercise Advice + NMES or Exercise Advice alone.

Patients at SET centres will first be offered the opportunity to attend the SET classes, and only if they do not wish to attend these classes will they be offered the opportunity to participate in the trial as a nonSET patient.

**Non-SET Centre**

- Control Group 2: Exercise Advice
- Treatment Group 2: Exercise Advice + NMES

**PRIMARY ENDPOINT:** Absolute walking distance (AWD) measured by treadmill testing at 3 months (the end of the intervention period).

**SECONDARY ENDPOINT(S):**

- Initial claudication distance (ICD)
- Quality of life – Intermittent Claudication Questionnaire (ICQ), EuroQoL 5D (EQ5D), Short Form 36 (SF-36)
- Haemodynamic assessment - Duplex ultrasonography, Laser Doppler Flowmetry (LDF), Ankle Brachial Pressure Index (ABPI)
- Health economic assessment
- Compliance with interventions
- Device experience questionnaire

**DEVICE:** Revitive IX (supplied by Actegy Ltd)

## background

### Peripheral Arterial Disease

Peripheral arterial disease (PAD) has a significant global health burden with over 202 million individuals suffering from a manifestation of this disease worldwide (1). Risk factors include smoking, advancing age, diabetes, dietary factors, high blood pressure, dyslipidaemia, lack of exercise and male gender (2). The impact of this burden is highly important as patients suffering from PAD are more likely to suffer co-morbid conditions related to an underlying atherosclerotic disease process, where-by narrowing or blockages of arteries supplying organs and limbs increase the risk of heart attacks and stroke (2).

### Intermittent Claudication

Intermittent claudication (IC) is the commonest manifestation of PAD, presenting as pain in the lower limbs on exertion, which settles after a period of rest. This has a significant impact on exercise tolerance and quality of life (QoL). Approximately 5-10% of the UK population are estimated to be suffering from IC (3).

### Treatment options for Intermittent Claudication

National guidelines from the National Institute for Health and Care Excellence (NICE) (4) recommend that all patients suffering from IC should receive first line treatment of best medical therapy (BMT) to control cardiovascular risk factors (which includes exercise advice), and supervised exercise therapy (SET). SET involves a number of lower limb related physical activities that are undertaken for a set period and duration under the supervision of a healthcare professional.

### Summary of current research

SETs are known to significantly improve the absolute walking distances in patients suffering from IC.

A Cochrane systematic review of the impact of SET on walking distances was carried out by Bendermacher et al in 2006 (5), and was repeated with updated study data in 2013 by Fokkenrood et al (6). The latter review included randomised controlled trial (RCT) data comparing SET to non- SET management in patients with IC. Fourteen studies with a total of 1002 participants were randomised and followed up for a duration between 6 weeks and 12 months, with the primary outcome measure being absolute walking distance (AWD) measured by treadmill testing. There was a significant improvement of AWD with a size effect of 0.69 (95% Confidence Interval (CI) 0.51-0.86) and 0.48 (95% CI 0.32-0.64) at 3 months and 6 months respectively. On average, there was an improvement in AWD of approximately 180 metres in the SET group (6).

There is a strong evidence base in favour of SET, contributing to the NICE recommended first line therapy strategy in all IC patients. Despite these benefits, SET remains underutilised in the UK. A postal audit published by Shalhoub et al in 2009 (7) showed that only 24% of 84 responding UK vascular surgeons had access to a SET for their IC patients.

An online survey audit was repeated in 2014 by Babber et al (8) as the first audit of SET access following the publication of the 2012 NICE guidance and was open to vascular surgeons in the UK and Ireland, as well as to members of a physiotherapy organisation as the main SET providers. This audit showed only 35% of 118 respondents had access to a SET. Where these programmes exist, the majority are run by qualified physiotherapists (85%) or specialist nurses (13%). The main reasons for lack of access were attributed to a lack of funding, staff and infrastructure. Where SET was available, compliance was a major concern with reasons including patient difficulties with travelling to the SET class, travel expenditure and time being cited. Therefore, the actual standard of care for 65% of the survey respondents reflecting generic practice in UK and Ireland is best medical therapy and exercise advice only. The cost of attending 2 hours of SET per week for a 3-month duration in a class of 10 patients is approximately £288 per patient, excluding the cost of travel and absence from work or other activities (9).

### Neuromuscular electrical stimulation

Electrical therapy for medical use has been practiced since the 18th century. Only in the last 30-40 years has there been an improvement in understanding of the optimal electrical parameters that can maximise clinical effect, however mass use of electrical therapy was largely limited by the ability to produce small, portable and affordable units. Within the last 20 years, advances in design technology have overcome this problem, making small, portable, affordable, safe and effective electrical therapy units readily available.

Neuromuscular electrical stimulation (NMES) is an emerging technology. A proof of concept pilot study of 20 patients with IC showed a significant improvement in AWD (102.3m vs. 187.2m, p<0.01), and both ‘disease specific’ and ‘generic’ QoL measures after using a commercially available NMES device for 6 weeks (10). Compliance as assessed by patient recorded diaries was 98.5% NMES device usage in the 6-week follow-up period.

A systematic review by Williams et al included 5 studies employing a number of different NMES devices used as a treatment strategy for patients with IC (11). Ninety-six patients with IC were compared between control and NMES groups in the 5 included studies showing up to 150% improvement in AWD at 4 weeks of intervention and 34% at 8 weeks.

### Mechanistic evaluation of the device

In addition to a robust assessment of the clinical efficacy of NMES in vascular disease, there is a lack of understanding of potential mechanisms of effective change that may be attributed to this form of intervention. Studies using intermittent pneumatic compression (IPC) on the calf and feet of patients suffering with IC have attempted to explain likely mechanisms for symptomatic improvement post intervention, and these may be similar for NMES. Delis et al suggest that the post compressive state of the calf muscle following IPC decreases the venous pressure therefore increasing the arterio-venous pressure gradient. This would then favour an increased arterial flow in order to stabilise the gradient mis-match (12). Measuring blood flow haemodynamics by duplex ultrasound (DU) has been utilised to evaluate the response of lower limb arteries to various interventions. Bemmelen et al (13) showed increased  popliteal artery blood flow up to 8 times on releasing a pneumatic cuff applied around the calf for 2 seconds in the seated position in healthy individuals. In patients with arterial disease, there was a mean 3-fold increase in blood flow. The suggested mechanism was an increase in the veno-arterial pressure gradient upon release of the cuff. In 10 healthy individuals using a Geko NMES device there was a significant increase in superficial femoral artery flow of 84% (P<0.005) (14). We intend to use DU to analyse the blood flow (cc/min) and time-averaged mean velocity (cm/s) in the superficial femoral artery of control patients at rest, in a seated position, as well as patients receiving the NMES device at rest, 15 minutes into use, 30 minutes into use, 1 minute after cessation and 5 minutes after cessation. A point 5 cm distal to the mid-inguinal point will be utilised and marked for accurate repeat measurements of the time-averaged mean velocity (TAMV) and blood flow at the same site at different time-points.

Laser Doppler flow (LDF) is an optical measurement technique using disruption of laser wavelengths delivered through skin-applied probes to measure skin microcirculation. In the study above, LDF significantly improved in 10 healthy patients using the Geko NMES device for 20 minutes (14). The same was not seen when using IPC. Continuous LDF will be used to assess change in perfusion at rest for the control group and throughout the device use, up to 5 minutes post cessation, in the intervention group.

The Revitive IX is a CE marked class IIa device that has the following pre-set ranges of electrical output parameters: Frequency 1 Hz – 50 Hz, Output current maximum 13 mA. The electrical intensity is variable and set by the user from 1 - 99 units. Therapeutic levels of intensity are dependent on an intensity sufficient to cause motor neurostimulation causing calf muscle contraction, whilst remaining comfortable for the user. Intensity level varies from person to person and may be dependent on factors including foot plantar surface skin hydration and comfort.

This footplate device provides a 30-minute pre-programmed session of electrical stimulation to the plantar aspect of the feet to activate the venous foot and calf muscles pumps. The user controls the intensity of the impulses, and therapeutic benefit is deemed to occur when impulses are sufficient to cause ankle flexion thereby activating the calf muscle and pump increasing venous return to the heart.

The device is designed to be used whilst the user is in a seated position, with the device placed on the floor. The user places bare feet, one on each foot pad; the device requires both feet to be on their respective foot pads in order to complete the electrical circuit and deliver the required energy to effect stimulation.

The default starting setting of the device is for a 30 minutes duration with intensity set at zero. The user is required to increase the intensity using the interface controls until the stimulation can be felt or the muscles in the feet and calves can be seen to contract and relax. The device will continue to provide stimulation until the timer reaches zero at which point the device will automatically switch off.

The user may stop the stimulation at any point by pressing the power button and turning the device off or when using the foot pads by lifting one foot from off the device to break the circuit.

The device is recommended to be used for 30 minutes per treatment session with a maximum total treatment time of 3 hours per day as required to minimise the potential for muscle fatigue.

Revitive IX is intended for use in the home setting without the need for involvement of a clinical professional and can be used for up to 3 hours daily in 30 minute sessions. The retail cost of the device is approximately £160.

The Revitive IX devices have been designed for use with home users in mind and so have been designed to provide simple operation with minimal instruction and without the need for involvement or supervision by a health care professional. The range of capabilities for typical home users can vary by a large degree and so it is highly desirable for the devices to be relatively simple and physically easy to use.

### Technological Basis of the Product

Revitive IX uses the principle of electrical muscle stimulation (EMS) to elicit its effect. The products deliver energy to the user in the form of an electrical current applied to the skin surface, which stimulates the sensory and motor nerves, producing muscle contraction.

The electrical current causes a temporary and local change of state of the nerve membrane polarisation resulting in the initiation of an action potential (impulse), which travels the length of the nerve to the innervated muscle fibres. In response a muscle action potential is generated and conducted along the muscle fibres causing the muscle to contract^1^

To be effective, stimulation has to be sufficiently large, of sufficient duration and of suitable shape to overcome the threshold for depolarisation of the membrane, otherwise no action potential will occur.

Successful nerve stimulation and muscle recruitment therefore depends on the intensity of current and the duration and the frequency of energy applied. However sufficient time is needed for membrane repolarisation (the recovery of the resting membrane potential after each action potential) and to allow muscle relaxation, metabolic recovery and to prevent premature accelerated fatigue. As such sufficient rest intervals also need to be incorporated within the stimulation pattern.

The manner in which the Revitive generates stimulation is described in detail in the current version of the Investigator’s Brochure.

### Clinical evaluation of NMES techniques and similar devices

Clinical evaluation of NMES techniques and similar devices resulted in the following:

28 papers (n=913) were identified that investigated the use of electrical stimulation (ES) to improve blood flow, circulation and swelling. These studies were conducted in a range of populations including;

- 17 studies in healthy individuals
- 4 in patients with diabetes
- 2 patients following knee surgery
- 1 study in each of the following; patients with amputation, investigating blood flow in patients with wounds, patients who had undergone hip / knee arthroplasty, patients with peripheral vascular disease and ankle sprain.

There were two studies (one in patients with diabetes and one in patients with wound healing) which used a healthy population as a control.

Please refer to Clinical Evaluation Report for full details of these studies.

Of the 28 studies (n=676) reporting a significant improvement in blood flow due to electrical stimulation, 19 (n=582) were deemed to be clinically significant. A range of electrical stimulation devices were used in the studies which showed that ES produced a clinically significant improvement in blood flow, with Revitive IX investigated in three studies and Duo-STIM and Health fit P4-Microstim devices all being used in two studies each.

In the 17 studies which investigated the effect of ES on blood flow in healthy population, the results of 13 studies were determined to be clinically significant (n=261). One study investigated the use of ES to reduce swelling during motionless standing. Post-test foot and ankle volume was significantly greater than pre-test volume after 30 minutes of motionless standing (t= -7.093, p<0.001), but there were no significant differences after 30 minutes of standing with NMES (t = -1.374, p<0.185). The mean volume changes from pre-test to post-test in the conditions without NMES and with NMES were significantly different (51 ±32 mL and 12 ±39 mL, respectively; t=3.905, p=0.001), and therefore NMES in healthy individuals appears to limit fluid accumulation in the lower limb. In a similar healthy volunteer study (n=49), there was a significant increase in calf blood flow following 4 hours NMES stimulation whilst seated, compared to the contralateral unstimulated control leg.

Three unpublished studies investigate the use of Revitive IX to improve blood flow and reduce swelling in healthy subjects as a means for comparison. The first study investigated electrical stimulation with different pulse widths on the peripheral tissue perfusion, comparing both wide pulse (1 ms, WPS) and narrow pulse (0.5 ms, NPS) stimulation. Both WPS and NPS enhanced tissue perfusion in calf and foot above resting baseline. At a current intensity of 20 mA both WPS and NPS enhanced perfusion in calf and foot tissues (calf p=0.001; foot p=0.014). A significantly higher intensity was needed with NPS compared to WPS to enhance and retain peripheral blood flow similar to exercise. Furthermore, WPS at current intensities of >15mA (RMS at 500Ω) produced larger joint movement amplitudes compared to NPS. The authors concluded that WPS requires smaller current intensity than conventional NPS to produce beneficial effects on peripheral tissue perfusion.

A second study with Revitive IX investigated the use of ES or exercise to alleviate lower limb swelling induced by a 40-minute session of prolonged inactivity. The authors report that 20 minutes of ES led to a significant (p=0.005) reduction in limb swelling, significant (p=0.003) increase in temperature of the foot, but not calf surface and significantly enhanced (p<0.0001) blood flux in both foot and calf. ES was shown to be at least or even more efficient as voluntary exercise in resolving circulation insufficiencies induced by prolonged inactivity.

The Revitive IX device was investigated (n=16) for its effects on foot and calf blood flow and tissue oxygenation compared to a voluntary exercise control. The data demonstrated that ES produced a 10-fold increase in blood flux above baseline in the foot (p<0.001) and 7-fold increase in the calf (p<0.001). In comparison, voluntary exercise produced 3-fold (p=0.008) and 2-fold (p=0.002) for foot and calf respectively. Furthermore, tissue oxygenation in the foot and calf significantly increased in the ES group compared to voluntary exercise (p<0.018).

The use of ES to increase the blood flow in patients with vascular disease (n=36) was investigated by Clover et al. They reported that the use of localised subcontractile electrical stimulation (3 h/day for 6 weeks) on the feet of patients with peripheral vascular disease led to a significant (p<0.05) increase in micro vessel density and tissue perfusion.

Of the 27 studies (n=897) which reported the use of ES in blood flow, circulation and swelling, only two commented on adverse events. One reported that there were no adverse events in the study, while the second reported local skin irritation at the electrode site which resolved after cleaning the electrode site 3 days post-operatively and changing the electrode.

The papers include a heterogeneous population with both healthy subjects (16/27) and a range of clinical presentations, some of whom would be expected to present with vascular impairment or a reason for lower limb swelling.

This includes three papers with data specific to the Revitive IX device demonstrating statistically and clinically significant outcomes in a healthy population. The papers have not been published, but this is in relation to commercial sensitivity and is normal in such circumstances. The effect of the stimulation (which was clearly significant) in these reports is predominantly achieved during the stimulation period.

Clinically, electrical stimulation is employed as a means to stimulate a local circulatory response for patients with peripheral vascular disease, persistent swelling of the limb, wounds and other similar conditions. The literature cited is a reasonable reflection of the range of available material, and the selection of papers represents a full non-biased reflection of outcomes.

The conclusion that electrical stimulation can have a significant effect on local circulation is generally supported by the literature. This effect is clearly most pronounced when the application is made at a motor level, though there is some evidence for circulatory changes even with sensory level stimulation (e.g. Wilkstrom et al 1999). This sensory level circulatory response appears to be 'real' and is a phenomenon which is under current investigation (Anzelmo and Watson, in process).

There are fewer papers presented which relate directly to an evaluation of swelling, though this is a fair reflection of the available evidence. Of the 4 cited papers, all appear to demonstrate a statistically and clinically significant change.

In terms of safety and adverse events reporting, not all papers make a specific comment in this regard (though this is now an essential reporting component of a modern publication). Two papers have made specific reference to adverse outcomes, though in fact only one (Jensen et al, 1985) reports an incident which was minor (skin irritation) and resolved within a short time frame. This would be consistent with the electrical stimulation literature across the whole range of applications. Thousands of stimulation procedures are routinely carried out in clinical practice and as a home treatment and the incidence of adverse effects reported (formally or otherwise) is very low. Assuming that those trials which make no comment with regards adverse events actually did not witness anything untoward, this means that of the 913 patients, only a limited number (not actually reported in the Jensen paper) reported minor skin irritation. This may well have actually been linked to the Betadine employed at the time of the surgery, though it cannot be ruled out as an irritation due to the stimulating electrodes. Even so, this is a very low incidence of a very minor 'adverse' effect which was easily resolved.

In Summary:

The claims made in relation to the effects of electrical stimulation on local circulation are supported by the cited literature.

- The cited literature is a fair reflection of the available material.
- The support for circulatory effects is stronger than the support for swelling effects, but this is largely due to the difference in the volume of published material covering these topics.
- The adverse effects reported are at a low incidence and are very minor in nature.

### Rationale for the study

Although some evidence of the efficacy of NMES in the management of patients with IC exists, there is a significant paucity of high quality research conducted in a powered and controlled fashion. The proposed study is vital to robustly identify the contribution of clinical change using NMES, compared to the current gold standard recommended practice of SET and, actual standard of care offered in the majority of the UK and Ireland, which is BMT (including exercise advice).

In addition, compliance with NMES is likely to be better as devices can be used in the patient’s own environment, at a time convenient to them for a variable duration unlimited by a SET programmed duration. The unit price of an NMES device is significantly less than SET and therefore if proven efficacious, this study may provide the necessary evidence required to effect a change in recommendations and policy for the benefit of patients.

Another aspect to the study is evaluating the potential underlying mechanism by which NMES may improve lower limb IC symptoms. A number of studies evaluating IPC have shown functional and symptomatic benefit in patients suffering from IC (11). Potential mechanisms include enhanced activation of the calf muscle pump increasing the venoarterial pressure gradient, thereby increasing the blood flow in the lower limbs. The drawback of IPC is expensive and bulky equipment, long duration of treatment required (3-4 hours daily) and discomfort of the pressures required to increase venous return. Lower limb NMES may mimic the effect of IPC by causing sufficient calf contraction to activate the calf muscle pump. Haemodynamic assessment whilst using the Revitive IX device has shown significant increases in lower limb arterial blood flow measured by ultrasonography in healthy individuals (16). Further haemodynamic assessment in a robust clinical trial of NMES in IC patients will help in advancing our understanding and assist in developing future technology to optimise the use of this mechanism for patient benefit.

Under the remit of the NIHR EME programme, this study will enable robust research to determine definitive clinical efficacy, mechanistic evaluation and cost effectiveness of a novel intervention that will significantly impact care provision and outcomes for patients with PAD.

If effective, NMES has the potential to improve flexibility and compliance to treatment with non-invasive therapies, reducing the burden of advancing disease on the NHS as fewer patients will require specialist care input or more invasive management. Adding a more accessible, safe and effective modality to the non-invasive management strategy for patients with intermittent claudication is of vital current importance with implementation of a readily available and clinically effective modernised tool providing a 21st century solution to an age old problem.

### Risk / Benefit Assessment

#### 1.10.1 Benefits

The device is expected to increase the walking distance in patients with intermittent claudication, and therefore have an adjuvant benefit on the same when provided in addition to supervised exercise programmes. It is also expected to cause a reduction in pain symptoms and reduced likelihood of major intervention in late stage PAD (critical limb ischaemia).

#### 1.10.2 Risks

The risks as defined in the Instructions for Use (IFU) and Investigators Brochure (IB).

## OBJECTIVES and endpoints

### Primary Objective

The research objective is to assess the clinical efficacy of a NMES device as an adjunct to the local standard care available at the study randomisation sites to improve walking distance in patients with IC. The number of sites is equally distributed between those that provide the NICE recommended gold standard of care for IC patients, including SET compared to real-world management as practiced by the majority of centres across the UK, including exercise advice only. The clinical efficacy will primarily be measured by a change in the Absolute Walking Distance (AWD) over the study protocol period, measured by a standardised treadmill test.

### Secondary Objective

In addition, secondary outcomes including validated QoL questionnaires and compliance data will assist in modelling for economic evaluation of this intervention compared to standard treatment practice in order to assess cost effectiveness.

Evaluation will also be undertaken with the objective to understand the underlying mechanisms for change in clinical and subjective outcomes in the form of lower limb gross and superficial haemodynamic assessment. This will be undertaken by using DU and LDF, respectively.

### Primary Endpoint

- Absolute walking distance (AWD) at 3 months– standardised treadmill test

### Secondary Endpoints

- Initial claudication distance (ICD)
- Quality of Life - Intermittent Claudication Questionnaire (ICQ), EuroQoL 5D (EQ5D-5L), Short Form 36 (SF-36)
- Haemodynamic assessment - Duplex ultrasonography, Laser Doppler Flowmetry (LDF), Ankle Brachial Pressure Index (ABPI)
- Health economic assessment
- Compliance with interventions
- Device experience questionnaire

## STUDY DESign

### Design

This is a multicentre randomised controlled study including 11 participating centres in England, equally distributed according to local therapy provision between supervised exercise therapy (SET) (6 centres) and exercise advice only (6 centres) with one centre, St George’s Hospital, acting as a dual site. Subject to any patient specific restrictions, all patients will also be provided with best medical therapy (BMT) in order to control for any cardiovascular risk factors.

### Treatment regimens

Subjects will be randomised to one of 2 treatments, depending on whether they are treated in a centre providing SET or not as shown below in Table 1.

| SET Centre Treatment* | Number of subjects | Treatment |
| --- | --- | --- |
| Control 1 | 48 | EA + SET |
| Treatment 1 | 48 | EA + SET + NMES |
| Total number of subjects SET centre | 96 |  |

| \| Non-SET Centre Treatment \| Number of subjects \| Treatment \| \| --- \| --- \| --- \| \| Control 2 \| 48 \| EA \| \| Treatment 2 \| 48 \| EA + NMES \| \| Total number of subjects non SET centre \| 96 \|  \| Total number of trial subjects \| 192 \| \| --- \| --- \|   Table 1. Summary of treatment allocation  *All patients will be expected to receive BMT as per local guidelines which includes exercise advice*  * Authorised SET centres will be permitted to randomise patients who do not wish to attend SET classes using a randomisation list that will allocate participants to Exercise Advice + NMES or Exercise Advice alone. |  |
| --- | --- | --- | --- | --- | --- | --- | --- | --- | --- | --- | --- | --- | --- | --- |
|  |  |
| **Figure 1: Study flow chart** | |


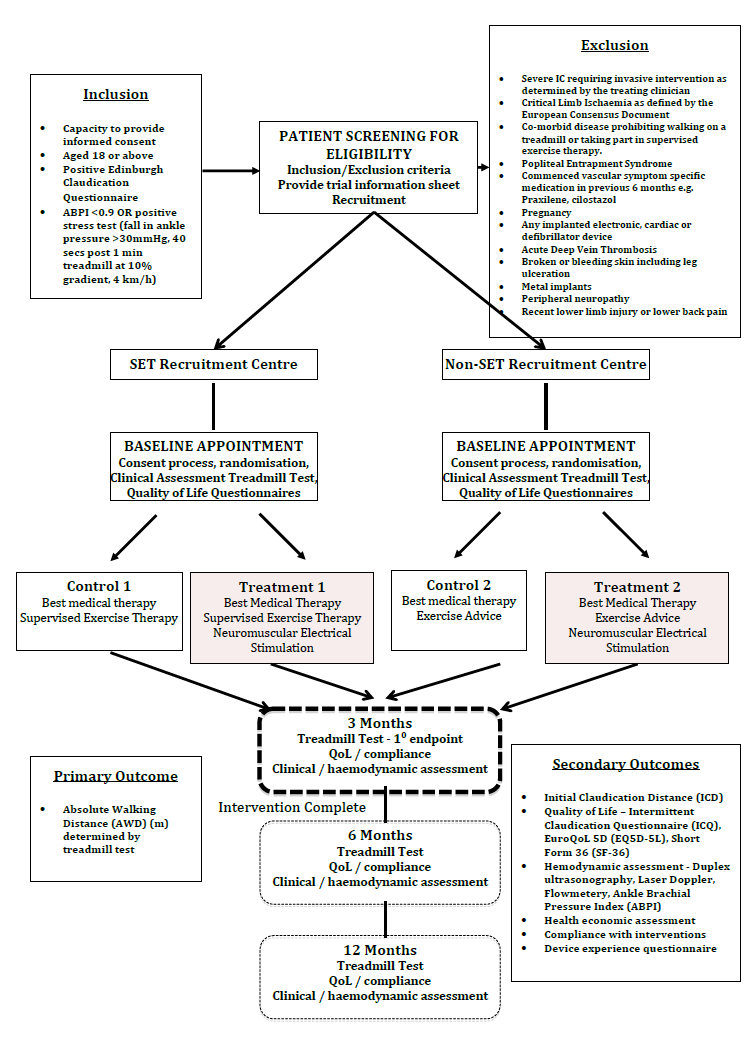


## PARTICIPANT ENTRY

### Study setting and population

This study is open to all patients at the participating NHS sites with a diagnosis of IC meeting specific inclusion and exclusion criteria.

The sites have been selected with their respect to their ability or not to provide SET.

The following six able to provide a SET programme^:

1. Imperial College Healthcare NHS Trust

2. North Bristol NHS Trust

3. Hull and East Yorkshire Hospitals NHS Foundation Trust

4. University Hospital Southampton NHS Foundation Trust

5. Dorset County Hospital NHS Foundation Trust

6. St George’s University Hospitals NHS Foundation Trust*

^Authorised SET centres will be permitted to recruit patients who do not wish to attend SET classes using a randomisation list that will allocate participants to Exercise Advice + NMES or Exercise Advice alone.

The following six are not able to provide a SET programme:

7. Cambridge University Hospitals NHS Foundation Trust

8. The Newcastle Upon Tyne Hospitals NHS Foundation Trust

9 .Taunton and Somerset NHS Foundation Trust

10. Nottingham University Hospitals NHS Trust

11. The Royal Bournemouth & Christchurch Hospitals NHS Foundation Trust

*a dual centre; a SET programme is only recommended in patients residing in the Wandsworth catchment area

#### 4.2 Inclusion criteria

- Capacity to provide informed consent
- Aged 18 or above
- Positive Edinburgh Claudication Questionnaire
- ABPI <0.9 OR positive stress test (fall in ankle pressure >30mmHg, 40 secs post 1 min treadmill at 10% gradient, 4 km/h)

#### 4.3 Exclusion criteria

- Severe IC requiring invasive intervention as determined by the treating clinician
- Critical limb ischaemia as defined by the European Consensus Document
- Co-morbid disease prohibiting walking on a treadmill or taking part in supervised exercise therapy.
- Able to walk for longer than 15 minutes on the study treadmill assessment
- Have attended supervised exercise therapy classes in the previous 6 months
- Popliteal Entrapment Syndrome
- Commenced vascular symptom specific medication in previous 6 months e.g. naftidrofuryl oxalate, cilostazol
- Pregnancy. Participants must be of non-childbearing potential* OR using adequate contraception for the duration of the study period and have a negative urine pregnancy test result
- Any implanted electronic, cardiac or defibrillator device
- Acute Deep Vein Thrombosis
- Broken or bleeding skin including leg ulceration
- Peripheral neuropathy
- Recent lower limb injury or lower back pain
- Already using a neuromuscular electrical stimulation device

* defined as those who have no uterus, ligation of the fallopian tubes, or permanent cessation of ovarian function due to ovarian failure or surgical removal of the ovaries. A woman is also presumed to be infertile due to natural causes if she has been amenorrheic for greater than 12 months and has an FSH greater than 40 IU/L

## PROCEDURES AND MEASUREMENTS

### Identification and recruitment of patients

Patients presenting with IC at the recruiting site vascular clinic will be identified by the direct healthcare team who will notify the research nurse or delegated individual to approach the participant with an information leaflet. This may be in person in the clinic, or by a patient invitation letter sent by mail / email or by telephone contact. Posters placed in participating research centres will support recruitment by signposting patients to the relevant research teams.

### Screening and pre-randomisation evaluations

Adults with IC presenting to vascular outpatient departments will be pre- screened by a member of the direct care team and invited to speak to a research nurse. With permission of the participant the reasons for non-inclusion will be logged anonymously along with a minimum data set of age, sex & ABPI and reason for exclusion. The anonymised pre-screening logs will be transferred to the Trial Coordinating Centre for the purposes of monitoring recruitment. Written informed consent will be obtained before the subject is enrolled in the study.

#### 5.2.1 Assessment of eligibility

**ABPI measurement <0.9:** The brachial blood pressure from the right arm using a manual blood pressure monitor cuff and Doppler will be recorded after 5 minutes of resting supine on a couch. The systolic blood pressure of the anterior tibial artery and posterior tibial artery at the ankle will also be recorded using the cuff and Doppler method. The ratio of the systolic brachial and ankle pressures will form the ABPI measurement.

OR

**Positive stress test:** (fall in ankle pressure >30mmHg, 40 secs post 1 min treadmill at 10% gradient, 4 km/h)

**Positive Edinburgh Claudication Questionnaire:** This defines a claudicant as someone who indicates pain in the calf, regardless of whether pain is also marked in other sites; a diagnosis of atypical claudication is made if pain is indicated in the thigh or buttock, in the absence of any calf pain. Subjects should not be considered to have claudication if pain is indicated in the hamstrings, feet, shins, joints or appears to radiate, in the absence of any pain in the calf.

**Medical & drug history & other criteria:** Review of inclusion and exclusion criteria including whether the patient has severe IC requiring invasive intervention, critical limb ischaemic, co-morbid disease prohibiting walking on a treadmill or taking part in SET. popliteal entrapment syndrome, commenced vascular symptom specific medication in previous 6 months e.g. naftidrofuryl oxalate, cilostazol, any implanted electronic, cardiac or defibrillator device, acute deep vein thrombosis, broken or bleeding skin including leg ulceration, peripheral neuropathy, recent lower limb injury or lower back pain

**Pregnancy test:** Participants must be of non-childbearing potential* (page 29) OR using adequate contraception for the duration of the study period and have a negative urine pregnancy test result

### Randomisation

Randomisation will take place via the InForm system (the electronic case report form database for the study), which will be programmed with a randomisation schedule provided by an independent statistician. Randomisation will be blocked with random block size and stratified by centres.

Once eligibility has been confirmed, subjects will be randomised to one of the two arms of the study and assigned a pseudononymised study number unique to each individual enrolled on the trial:

**SET Centre randomisation:**

- Best medical therapy including exercise advice and supervised exercised (control 1) OR
- Best medical therapy including exercise advice, supervised exercised and neuromuscular electrical stimulation (treatment 1)

Authorised SET centres will be permitted to randomise patients who do not wish to attend SET classes (i.e. nonSET patients). Therefore, the authorised SET sites will act, for the purposes of these participants, as nonSET sites. In doing so they are still following the basic protocol instructions in that they are providing best medical therapy, as agreed between patient and clinicians, which in this case is not to participate in the SET classes.

At these centres, the InForm database will be linked to a SET and nonSET randomisation list. The appropriate randomisation list will be used by sites dependent on whether the patient is attending / not to attending the SET programme.

**Non SET Centre randomisation:**

- Best medical therapy including exercise advice (control 2) OR
- Best medical therapy, including exercise advice and neuromuscular electrical stimulation (treatment 2)

### Other baseline investigations

- **Demographic information & vital signs:** age, ethnicity, date of birth, lifestyle, socioeconomic status, weight, height, blood pressure, pulse
- **Treadmill:** The Gardner-Skinner graded treadmill test will be used (15). The treadmill will start at 3.2 km/h at a 0% incline. Every 2 minutes, the gradient of the treadmill will increase by 2%. Patients will indicate the start of their claudication pain, which will be recorded as the Initial Claudication Distance (ICD) and finally stop the test at the point the patient does not want to continue due to lower limb pain; this is the Absolute Walking Distance (AWD). The patient will not be given a final score to prevent bias. If patients are able to walk for longer than 15 minutes on the treadmill, they will be excluded from the trial.
- **Quality of Life questionnaires:** The validated EuroQoL (EQ-5D-5L) questionnaire and Short Form-36 (SF-36) questionnaire will be used to assess the generic QoL and will allow economic assessment based on intervention and score improvement. The validated Intermittent Claudication Questionnaire (ICQ) will be used to assess change in disease-specific QoL. These should be completed prior to informing the participant of the treatment allocation to prevent bias.
- **Haemodynamic assessment**
- **Duplex ultrasonography:** Using the appropriate arterial ultrasound probe and pre-set volume flow algorithms on a duplex ultrasound machine, flow within the common femoral artery (CFA), preferably of the most affected limb, will be measured, approximately 5cm below the mid-inguinal point at the groin. Measures include volume flow (VF, cc/min) and time-averaged mean velocity (TAMV, cm/s).
  - **In the control group**: only resting values will be undertaken over a 3-minute period.
  - **The intervention group** will have these parameters measured at rest, at 15 and 30 minutes into device use and then at 1 and 5 minutes after device cessation (16).
- **Laser Doppler flowmetry** - The single fibre laser optical probe of the LDF machine will be positioned on the dorsal aspect of the foot using single use adhesive pads. The LDF device measures skin surface temperature and superficial skin perfusion or blood flux, as a measure of superficial skin blood flow. Once the probe is placed, measurement is continuously collected via the LDF software.
  - **In the control group:** this will be at rest for a 3 minutes duration
  - **The intervention group:** data will be collected at rest until 5 minute after device cessation.

### Treatment

There will be no change to the local site standard of care for patients with IC attributed to participation in this trial. Those sites with SET will continue to provide this intervention as per their normal standard of care and locally agreed protocol. The treatment period will last for 3 months.

#### 5.5.1. Best medical therapy

Best medical therapy for PAD (comprising of smoking cessation, antiplatelet agent use, cholesterol reduction, exercise therapy, and the diagnosis and treatment of hypertension and diabetes mellitus) will be as per standard care and local guidelines and it not standardised by this protocol.

#### 5.5.2. Exercise advice

The participants will be given standard advice on exercise as per local guidelines. Participants will be provided with a diary to note the frequency and duration of their exercise activity.

#### 5.5.3. Supervised exercise programme (SET centres only)

The supervised exercise programme will be as per standard care and local guidelines and is not standardised by this protocol but usually involves at least 30 minutes of physical activity made up of rotating through various low impact exercises (treadmill walking, steps, stretching etc.) under the supervision of the programme leader as per the NICE Guidance 147: Lower limb peripheral arterial disease. August 2012. <http://guidance.nice.org.uk/CG147> .

The SET will be carried out under the supervision of a healthcare professional and entail a circuit of lower limb specific exercises for a minimum of 30 minutes per week usually over 3 months duration but as per local policy and attendance at SET classes will be recorded. This programme should occur at least once per week, however patients will be advised to continue 30 minutes of daily exercise to similar intensity between these sessions in line with the Department of Health and Chief Medical Officer's guidance on daily physical activity to promote a healthier lifestyle and prevent disease.

#### 5.5.4. Neuromuscular electrical stimulation device

Revitive IX is a disc shaped, footplate device used in a seated position with the soles of both feet in contact with the conductive footpads. The user is able to alter the stimulation intensity until there are visible strong yet comfortable calf muscle contractions and associated heel-toe raises.

The groups randomised to the NMES device, will be familiarised with the device by asking them to place the soles of both feet onto the respective footpads. The IsoRocker will be enabled throughout the study period. Baseline sensory and motor thresholds will be established and noted. The sensory (perception) and motor thresholds will be established by systematically varying the stimulation intensity with steps of ±1% maximal output whilst the participant provides verbal sensory feedback. The minimal intensity, which the participant is able to clearly feel, will be recorded as the perception threshold, and that producing visible muscle twitches as the motor threshold. The stimulation intensity for the main interventions will be adjusted to produce visible strong but non-painful contraction of the lower limb musculature based on the individual suprathreshold. Participants will be instructed to use the device to as close a stimulation intensity to this suprathreshold level without the experience being uncomfortable. Participants will be made aware that they may need to increase the intensity over time to compensate for habituation (familiarisation with the electrical muscle stimulation).

Patients will be advised to complete at least one pre-programmed 30-minute session of NMES daily, to a maximum of 6 sessions and record usage in the compliance diary. Where patients are also diagnosed with diabetes, a minimum usage of two (2) x 30 minute sessions per day is recommended. In addition, some devices will be fitted with a voltage/current data logger which will connect between the mains supply and the device, recording usage each time. This information will be recorded on the data logger memory and uploaded following the 3 months intervention period in order to cross reference against the compliance diary. This will act as a further robust mechanism to capture adherence to device use, with measurement of frequency of device use.

### Follow-up

Follow-up data will be collected at 3 months, 6 months and 12 months. These visits should be performed at the study site to allow the treadmill test, ABPI / Peripheral pulse, haemodynamic assessments to take place, however, in the event that the participant is unable to attend in clinic or the site is unable to accommodate the on-site visit these visits may take place remotely (i.e., over the telephone completely or in combination with postal questionnaires). These visits should only take place remotely if this has been previously discussed and agreed with the Trial Coordinating Centre. Missed assessments as a result of a remote visit should be re-scheduled at a later date as a separate on-site visit, where possible.

Sites should clearly document the mode of follow-up that takes place, such as face to face or over the telephone.

#### 5.6.1. 3 Month Visit (end of treatment phase)

Assessments and data collection will be collected as follows:

- **Treadmill test as per baseline**
- **ABPI / Peripheral pulse as per baseline**
- **Drug history review**
- **Safety reporting**
- **Quality of Life questionnaires as per baseline**
- **Haemodynamic assessment as per baseline**
- **Duplex ultrasonography:**
  - **In the control group**: only resting values will be undertaken over a 3-minute period.
  - **The intervention group** will have these parameters measured at rest, at 15 and 30-minutes into device use and then at 1 and 5 minutes after device cessation.
- **Laser Doppler flowmetry**:
  - **In the control group:** this will be at rest for a 3 -minute duration
  - **The intervention group:** data will be collected at rest until 5 minutes after device cessation.
- **Compliance and resource diaries**

1. **Exercise compliance -** Participants will be given a compliance diary to complete attendances at the SET (if SET centre), and record the type and frequency of exercise undertaken in their own time during the study period.
2. **Resource diary -** Participants will be given a resource diary to complete health care resource use during the study period.
3. **Device compliance & experience (device arm only) -** Participants randomised to use the NMES device will be provided with a compliance diary to record device use details. In addition, information from the data logger (if applicable) will be assessed in order to cross reference with the compliance diary. At 3 months, a simple device use questionnaire will be taken to report ease of device use and suggest any developments.

Patients will receive weekly text messages during the treatment phase to remind them to complete their diaries, and attend SET or follow exercise advice.

In the event that the participant is unable to attend in clinic or the site is unable to accommodate the on-site visit, this visit may take place remotely (i.e. over the telephone completely or in combination with postal questionnaires). This visit should only take place remotely if this has been previously discussed and agreed with the Trial Coordinating Centre. Missed assessments as a result of a remote visit should be re-scheduled at a later date as a separate on-site visit, where possible.

Sites should clearly document the mode of follow-up that takes place, such as face to face or over the telephone.

#### 5.6.2. 6 Month Visit

Assessments and data collection will be collected as follows:

- **Treadmill test as per baseline**
- **ABPI / Peripheral pulse as per baseline**
- **Drug history review**
- **Safety reporting**
- **Quality of Life questionnaires as per baseline**
- **Haemodynamic assessment**
- **Laser Doppler flowmetry only (no duplex)**: this will be at rest for a 3-minute duration in the most affected leg.
- **Compliance and resource diaries**

1. **Exercise compliance -** Participants will be given a compliance diary to complete attendances at the SET if it runs past the 3-month visit (if SET centre), and record the type and frequency of exercise undertaken in their own time during the study period.
2. **Resource diary -** Participants will be given a resource diary to complete health care resource use during the study period.

In the event that the participant is unable to attend in clinic or the site is unable to accommodate the on-site visit, this visit may take place remotely (i.e. over the telephone completely or in combination with postal questionnaires). This visit should only take place remotely if this has been previously discussed and agreed with the Trial Coordinating Centre. Missed assessments as a result of a remote visit should be re-scheduled at a later date as a separate on-site visit, where possible.

Sites should clearly document the mode of follow-up that takes place, such as face to face or over the telephone.

#### 5.6.3. 12 Month Visit

Assessments and data collection will be collected as follows:

- **Treadmill test as per baseline**
- **ABPI / Peripheral pulse as per baseline**
- **Drug history review**
- **Safety reporting**
- **Quality of Life questionnaires as per baseline**
- **Haemodynamic assessment**
- **Laser Doppler flowmetry only (no duplex)**: this will be at rest for a 3minute duration in the most affected leg.
- **Compliance and resource diaries**

1. **Exercise compliance -** Participants will be given a compliance diary to complete attendances at the SET if it runs past the 3-month visit (if SET centre), and record the type and frequency of exercise undertaken in their own time during the study period.
2. **Resource diary -** Participants will be given a resource diary to complete health care resource use during the study period.

In the event that the participant is unable to attend in clinic or the site is unable to accommodate the on-site visit, this visit may take place remotely (i.e. over the telephone completely or in combination with postal questionnaires). This visit should only take place remotely if this has been previously discussed and agreed with the Trial Coordinating Centre. Missed assessments as a result of a remote visit should be re-scheduled at a later date as a separate on-site visit, where possible.

Sites should clearly document the mode of follow-up that takes place, such as face to face or over the telephone.

The 12-month follow up appointment will mark the end of the study participation.

### Summary of Visit Schedule

|  | **Screening** | **Baseline*** | **Treatment Phase** | **Follow-up (months)†** | | |
| --- | --- | --- | --- | --- | --- | --- |
| **Visit** | **1** | **2** |  | **3** | **4** | **5** |
|  |  |  | **0 to 3 months** | **3 months** | **6 months** | **12 months** |
| Informed consent | **X** |  |  |  |  |  |
| Pregnancy test^1^ | **X** |  |  |  |  |  |
| Ankle Brachial Pressure Index (ABPI) / positive stress test | **X** |  |  | **X** | **X** | **X** |
| Edinburgh Claudication Questionnaire | **X** |  |  |  |  |  |
| Medical history | **X** |  |  |  |  |  |
| Drug history | **X** |  |  | **X** | **X** | **X** |
| Peripheral pulse examination | **X** |  |  | **X** | **X** | **X** |
| Other exclusion criteria | **X** |  |  |  |  |  |
| Randomisation |  | **X** |  |  |  |  |
| Demography |  | **X** |  |  |  |  |
| Vital signs |  | **X** |  |  |  |  |
| EQ-5D-5L / SF-36 / ICQ |  | **X** |  | **X** | **X** | **X** |
| Treadmill test (ICD/ AWD) |  | **X** |  | **X** | **X** | **X** |
| Duplex Ultrasonography |  | **X**^2^ |  | **X**^2^ |  |  |
| Laser Doppler Flowmetry |  | **X**^2^ |  | **X**^2^ | **X** | **X** |
| NMES training^3^ |  | **X** |  |  |  |  |
| SET booking^4^ |  | **X** |  |  |  |  |
| Compliance Diary (SET / device / EA) |  | **X** |  | **X** | **X** | **X** |
| Resource use diary |  | **X** | **X** | **X** | **X** | **X** |
| Weekly text messages |  |  | **X** |  |  |  |
| Data logger |  | **X** | **X** |  |  |  |
| Device experience questionnaire |  |  |  | **X** |  |  |
| Safety reporting |  |  | **X** | **X** | **X** | **X** |

**Baseline and screening visit occur on the same day if both the researcher and participant agree that informed consent has been adequately considered with time to ask questions*

*† These visits may take place remotely In the event that the participant is unable to attend in clinic or the site is unable to accommodate the on-site visit*

*^1^women of child bearing potential a required to take a urine pregnancy test*

^2^ *At rest and during device use in device arm*

^3^ *NMES treatment groups only*

^4^*SET centres only where applicable*

## TREATMENTS

### Investigational Medical Device Details

This trial is being carried out under a MHRA Devices (Notice of No Objection). The device is therefore only to be used by the named investigators, for the participants specified in this protocol, and within the trial.

#### 6.1.1. Description of the Device

The Revitive IX System comprises of the following Components:

**EMS Device: REVITIVE IX:**

C: Foot Pads

D: LED Display Panel

E: Time Setting Controls

F: Foot pad Intensity Controls

G: Electrode Pad Intensity Controls

H: Power Button

I: Location of Accessory and Power Sockets

J: IsoRocker

**AC Power Adaptor:**

The AC/DC Power adaptor is a CE Marked Class II Portable AC Adaptor with a rated input of 100-240VAC, 50-60Hz, 0.18A and a Rated Output of 5.0VDC, Max 1.0A. The adaptor is certified to EN60601-1:2006 by TUV SUD Product Service GmbH. The AC adaptor is designed and manufactured in accordance with a Quality Management Systems certified to ISO 9001:2008.

**Remote Control:**

The IR remote control is operated by 2 x 1.5v AAA batteries (supplied, not installed) and interacts with the digital display located on the main unit of Revitive Medic where the receiver is located. The remote control provides duplicate functions to the user interface on the Revitive Medic device. The Remote Control is designed and manufactured in accordance with a Quality Management System certified to ISO 9001:2015.

The Revitive IX system consists of an electrical muscle stimulation (EMS) device, AC/DC power adaptor and optional electrode pads. EMS is the application of electrical impulses that are of sufficient intensity to produce an artificial contraction of the muscle tissue. Revitive IX is indicated for electrical stimulation of the lower leg to:

- Reduce swelling in the legs/ankles/feet - caused by being immobile due to osteoarthritis or an injury
- Improve circulation to reduce or prevent blood-pooling (stasis) - caused by diabetes or by being immobile following surgery
- Reduce pain and discomfort in the legs/ankles/feet - caused by diabetic peripheral neuropathy or swelling (oedema) due to osteoarthritis or following surgery
- Increase muscle strength to help regain mobility in the legs affected by being immobile due to COPD, osteoarthritis or following surgery
- Help maintain leg vein health - by increasing circulation, delivering more oxygenated-blood and reducing swelling (oedema) in the legs/feet/ankles.

In addition to the medical indications the Revitive family of devices are intended to provide electrical muscle stimulation for the following:  Healthy individuals who have a sedentary lifestyle or spend long periods inactive, Revitive may help to:

- Reduce swollen feet and ankles
- Alleviate tired, aching & heavy legs, including cramp
- Improve circulation
- Help maintain leg vein health
- Increase muscle strength in the legs

The device is intended for home use for a recommended a minimum of 30 minutes treatment, up to a maximum of 3 hours treatment time per day as required. The device delivers electrical stimulation to the lower limb muscles through foot pads and/or through electrode pads which may be positioned on the lower limb.

The Revitive IX provides user control of intensity and duration of stimulation. It also has an additional IsoRocker feature that allows ankle movement during stimulation. When enabled, the IsoRocker allows Revitive IX to tilt back and forth as the muscles contract and relax. The user is able to choose whether to enable the IsoRocker feature on the device. Revitive IX also includes a remote control in addition to the controls on the device, and Revitive IX is reusable. It is supplied non-sterile with an AC adaptor, remote control (including 2 x 1.5v AAA batteries) and electrode pads as standard.

The technical characteristics of the Revitive IX device is summarised in the current version of the current version of the Investigator’s Brochure.

#### 1.6.2. Details concerning the manufacturer of the investigational device.

All of the manufacturing operations for the routine production of Revitive IX devices are subcontracted through an approved supplier comprising:

Mirae Medi & Tech Co. Limited

Full details are provided in Revitive IX Technical File Section Principal Subcontractors.

The manufacture of Revitive IX is carried out in line with documented production and inspection procedures.

An overview of the manufacturing process and details of the quality assurance checks are provided in the in the current version of the Investigators’ Brochure.

**1.6.3. Name or number of the model/type, including software version and accessories, if any to permit full identification:**

Name: Revitive IX Model: RIX Ref: 1379 Software Version: 2.0

**1.6.4. Description as to how traceability shall be achieved during and after the clinical investigation:**

Traceability is achieved by assignment of device serial numbers to patient identifiers.

**1.6.5. Intended purpose of the investigational device in the proposed clinical investigation**

Revitive IX devices are intended for electrical stimulation of the lower leg.

**1.6.6. Populations and indications for which the investigational device is intended**

The Revitive IX device is intended for electrical stimulation of the lower leg in healthy individuals.

The device is intended for home use for one 30 minutes treatment per day, up to no more than 3 hours per day

**1.6.7. Summary of necessary training and experience needed to use the investigational device**

Revitive IX has been designed for use with home users in mind. The range of capabilities for typical home users can vary by a large degree and so it is highly desirable for the device to be relatively simple and physically easy to use. As such the device has been designed to provide simple operation with minimal instructions as provided in the instruction sheet accompanying the device.

The research team conducting the study, and study participants, will be instructed in the use of the device as follows:

- How to switch on and off
- How to place feet on the footpads and set intensity and duration
- How to clean the device
- How to use the remote control
- How to operate the IsoRocker feature

#### 1.6.8. Patient Compliance Monitoring

The investigational device will be fitted with a data logger which will monitor the device power input at the connection to the mains power adaptor and will provide a trace of usage during the study period. This data will be utilised to assess patient compliance to the treatment regime. Patients will also be required to maintain a diary throughout the duration of the trial, and they will be sent text message reminders to do so.

**1.6.9. Principle of Operation**

NMES^22^ is the application of electrical impulses, which are of sufficient intensity to produce an artificial contraction of the muscle tissue. Through the introduction of electrical stimulation to the muscles of the feet and legs, the muscles are made to repeatedly contract and relax, restoring the pumping action and thereby helping to counteract stasis, increase blood flow and reduce swelling.

Revitive IX is a Class IIa active medical device intended for transient use, in accordance with AnnexIX, Rule 9 of EC Council Directive 93/42/EEC concerning medical devices as amended by Directive 2007/47/EC: “*All* *active* *therapeutic* *devices* *intended* *to* *administer* *or* *exchange* *energy* *are* *in* *Class IIa”*

### Labelling and Packaging

In accordance with Medical Devices Directive: 93/42/EEC (as amended by Directive 2007/47/EC) all Revitive IX devices intended for clinical investigation must bear the wording "exclusively for clinical investigation".

It is recognised that such wording may cause confusion to clinical staff in that it may be thought that the clinical investigation being referred to is of a patient rather than the device. Local Principal Investigators should ensure that the meaning of this wording is clearly understood by all staff using or coming into contact with the device being investigated and that the device under investigation is segregated, where possible, from any similar devices in routine use.

### Storage

The device should be stored as per the instructions for use.

### Permanent Discontinuation of Study Treatment and Withdrawal from Study

#### Permanent discontinuation of study treatment

Subjects may discontinue study treatment for the following reasons:

- At the request of the subject.
- Serious Adverse Event
- If the investigator considers that a subject's health will be compromised due to adverse event or concomitant illness that develop after entering the study.

#### Withdrawal from Study

Withdrawal from the study refers to discontinuation of study treatment and study procedures and can occur for the following reasons:

- Subject decision
- Loss to follow-up
- Subject loss of capacity

#### Procedures for Withdrawal from Study

There are no criteria for withdrawal from the study. Patients will be free to withdraw from the study without any effect on their usual medical care. The reason for their withdrawal will be recorded in the CRF/eCRF and medical records if offered. All randomised will be followed up to 12 months unless they specifically asked to be withdrawn as per intention to treat. In line with this analysis patients lost to follow up or withdrawn from the study will not be replaced.

#### Procedures for Withdrawal from Study due to loss of capacity

If a patient loses capacity after consenting to take part in the study, the local Principle Investigator may decide it is in the patient’s best interests to be withdrawn. Any identifiable data already collected with consent will be retained and may be analysed, but no further data will be collected or any other research procedures carried out on or in relation to the patient.

## SAFETY REPORTING

### Adverse Events

### Adverse Event Definition

**Adverse Event (AE)** An AE is any untoward medical occurrence, unintended disease or injury or any untoward clinical signs (including an abnormal laboratory finding) in subjects, users or other persons whether or not related to the investigational medical device.

- This includes: events related to the investigational device or the comparator
- Includes, events related to the procedures involved (any procedure in the protocol)
- For users or other persons this is restricted to events related to the investigational medical device

### Adverse Event Recording

For the purposes of the study, all AEs will be followed up according to local practice until the event has stabilised or resolved, whichever the sooner is. It is essential that all AEs that occur during the course of the study are appropriately reported in order to ensure the participants continuing safety. Of particular importance is the assessment of any event for *causality* and *expectedness* in relation to the device.

### Severity of Adverse Events

Mild: Awareness of event but easily tolerated

Moderate: Discomfort enough to cause some interference with usual activity

Severe: Inability to carry out usual activity

### Causality of Adverse Events

- Unrelated: No evidence of any causal relationship between the use of the device and the AE.
- Unlikely: There is little evidence to suggest there is a causal relationship with the use of the device (e.g. the event did not occur within a reasonable time after study treatment). There is another reasonable explanation for the event (e.g. the patient’s clinical condition, other concomitant treatment).
- Possible: There is some evidence to suggest a causal relationship between the use of the device and the AE (e.g. because the event occurs within a reasonable time after study treatment). However, the influence of other factors may have contributed to the event (e.g. the patient’s clinical condition, other concomitant treatments).
- Probable: There is evidence to suggest a causal relationship between the use of the device and the AE and the influence of other factors is unlikely.
- Definite: There is clear evidence to suggest a causal relationship between the use of the device and the AE and other possible contributing factors can be ruled out.

### Serious Adverse Event (SAE)

- - 1. **Serious Adverse Event Definition**

**Serious Adverse Event (SAE)** is any adverse event that has

1. Led to a death
2. Led to a serious deterioration in health that either:
3. Resulted in a life-threatening illness or injury, or
4. Resulted in a permanent impairment of a body structure or a body function, or
5. Required in-patient hospitalisation or prolongation of existing hospitalisation, or
6. Resulted in medical or surgical intervention to prevent life threatening illness or injury or permanent impairment to a body structure or a body function
7. Led to foetal distress, foetal death or a congenital abnormality or birth defect

This includes device deficiencies that might have led to a serious adverse event if a) suitable action had not been taken or b) intervention had not been made or c) if circumstances had been less fortunate. These are handled under the SAE reporting system.

A planned hospitalisation for pre-existing condition, or a procedure required by the protocol, without a serious deterioration in health, is not considered to be a serious adverse event.

#### 7.6.2. Serious Adverse Event Reporting

If an AE is assessed as serious, the PI or designated site staff member will complete the SAE form on the InForm database immediately or within 24 hours of being made aware of the event. In cases where the InForm system is unavailable, sites will email the completed paper SAE form to the coordinating centre via [nesictrial@imperial.ac.uk](mailto:nesictrial@imperial.ac.uk) immediately or within 24 hours of being made aware of the event. All SAEs will be reported to the JRCO as soon as possible after becoming aware of the event.

SAEs which indicate an imminent risk of death, serious injury or serious illness and require prompt remedial action for other patients, users or other persons or a new finding to it, must be reported to the MHRA by the Sponsor immediately but no later than 2 calendar days following the date the Sponsor is made aware.

Any other reportable events should be reported immediately but no later than 7 calendar days following the date the Sponsor is made aware.

The device manufacturer should also be informed within 24 hours of the SAE or device deficiency (if indicated in the study’s communication agreement).

<http://www.mhra.gov.uk/Safetyinformation/Reportingsafetyproblems/Devices/index.htm>.

### Adverse Device Effect (ADE)

#### 7.7.1. Adverse Device Effect Definition

**Adverse Device Effect** is an adverse event related to the use of an investigational medical device.

Includes any adverse event resulting from insufficiencies or inadequacies in the instructions for use, the deployment, the implantation, the installation, the operation, or any malfunction of the investigational medical device

Includes any event that is a result of a use error or intentional misuse

(Note: All AEs judged by either the reporting investigator or the Sponsor as having a reasonable causal relationship to the device qualify as adverse effects)

#### 7.7.2. Anticipated Adverse Device Effects

Full details of risks and anticipated adverse device effects (ADEs) for REVITIVE are contained within the Risk Management Report for REVITIVE® Electronic Muscle Stimulators (EMS), report reference: RMR/RIX/007 (document title “7. RMR_RIX_v7.0 Combined Signed”).

#### Recording Adverse Device Effects

An adverse event defined by the PI as an adverse device effect should follow the same recording procedures as AEs (defined in section 7.3). ADEs form part of the ongoing safety review for the study and are not classified as reportable to either the MHRA or manufacturer, unless specified in the communication agreement.

### Serious Adverse Device Effects (SADE)

- - 1. **Serious Adverse Device Effect Definition**

An adverse device effect that has resulted in any of the consequences characteristic of a serious adverse event or that might have led to any of these consequences if suitable action had not been taken or intervention had not been made or if circumstances had been less opportune. SADEs can be classified into either Anticipated Serious adverse effects (ASADE) or unanticipated serious device effects (USADE)

### Anticipated Serious Adverse Device Effects (ASADE)

#### 7.9.1. Anticipated Serious Adverse Device Effect Definition

A serious adverse device effect which by its nature, incidence, severity or outcome has been previously identified in the risk analysis report or Clinical Investigation Brochure.

#### 7.9.2. Reporting Anticipated Serious Adverse Device Effects

The PI or designated site staff member will complete the SAE form on the InForm database immediately or within 24 hours of being made aware of the event. In cases where the InForm system is unavailable, sites will email the completed paper SAE form to the coordinating centre via nesictrial@imperial.ac.uk immediately or within 24 hours of being made aware of the event.

SAEs which indicate an imminent risk of death, serious injury or serious illness and require prompt remedial action for other patients, users or other persons or a new finding to it, must be reported to the MHRA by the Sponsor immediately but no later than 2 calendar days following the date the Sponsor is made aware.

Any other reportable events should be reported immediately but no later than 7 calendar days following the date the Sponsor is made aware.

The device manufacturer should also be informed within 24 hours of the SAE or device deficiency (if indicated in the study’s communication agreement).

### Unanticipated Serious Adverse Device Effects (USADE)

#### 7.10.1. Unanticipated Serious Adverse Device Effect Definition

A serious adverse device effect which by its nature, incidence, severity or outcome has not been identified in the current version of the risk analysis report, Instructions For Use or Clinical Investigation Brochure.

#### Reporting Unanticipated Serious Adverse Device Effects

In addition to reporting to the MHRA (as detailed in the SAE section), the Sponsor will report the USADE to the REC within 15 days. The Sponsor will also notify the Device Manufacturer and Investigators at all sites of the USADE.

Follow up of patients who have experienced a USADE should continue until recovery is complete or the condition has stabilised.

### Developmental Safety Update Reports / Annual Safety Reports

Annual Safety reports will be submitted to the Sponsor, the Ethics Committee and Regulatory Authority in accordance with regulatory requirements.

### Pregnancy

Where relevant, any pregnancy occurring during the clinical study and the outcome of the pregnancy should be recorded on a pregnancy notification form patients will be asked to consent to be followed up for congenital abnormality or birth defect. Pregnancy is considered an SAE, the patient will be asked to stop using the device, followed up according to the protocol and the results analysed as per intention to treat.

### Reporting urgent safety measures

If any urgent safety measures are taken the CI/Sponsor shall immediately and in any event no later than 3 days from the date the measures are taken, give written notice to the MHRA and the relevant REC of the measures taken and the circumstances giving rise to those measures.

## STATISTICAL ANALYSES

### . Sample size and power considerations

This will be a two-arm randomised controlled trial, where the control is locally available therapy, which includes Best Medical Therapy AND either exercise advice or SET depending on the centre. The intervention, in addition to the locally available therapy, is NMES.

Four studies have reported on improvement in absolute walking distance (AWD) for the same population of patients (17-20). Three studies report AWD at 3 months, and for these improvement varied between 75-90 metres (18-20). The MIMIC trial does not report the effect size at 3 months (17). The improvement detected at 6 months varied from 41m (17) to 170m (18) across all studies. None of the studies clearly reported the standard deviation associated with the treatment effect measurement. The MIMIC trial (17) assumed a standard deviation (SD) of 120m for an effect size of 60m in their sample size calculation.

From pilot work and clinical judgement, using NMES together with best locally available therapy should at least result in an improvement of 60m after 3 months in the intervention group mean AWD. This improvement is deemed as clinically important. Therefore, we have adopted the same assumptions of effect size and standard deviation for the sample size calculation for our study. These parameters are deemed as providing significant clinical benefit in the IC population translating in improvement of lifestyle factors as measured by the QoL questionnaires.

Assuming that the mean AWD in the control group is 200m at 3 months (18) and a common equal standard deviation of 120m (17), without considering the dropout rate, it is estimated that a sample size of 172 participants (86 per group) will have 90% power with a two sided alpha = 0.05 to detect a difference of 60m in the mean absolute walking distance at 3 months between the intervention and the control group.

Assuming a 10% dropout rate, the sample size required for this study is 192 participants. The sample size was computed for a two-sample means test using Stata 13.

### . Planned recruitment rate

To recruit sufficient subjects 11 centres will participate in the study: six centres with exercise advice only as locally available therapy with the remainder providing supervised exercise therapy.

Through feasibility data collected from each centre, in total, 24 patients with IC could theoretically meet inclusion criteria for randomisation per month.

Accounting for 4 centres, which also undertake other studies with the same patient population, we anticipate randomising 13 patients per month; this would provide an adequate number of subjects. Therefore, a recruitment period of 15 months would be required to reach the target recruitment of 192 patients for this study.

Due to a variation in size of site, numbers of patients with Intermittent Claudication seen and involvement in other studies, the recruitment targets per site will be varied but we will ask sites to aim to recruit 24 patients in total (2 per month) for 15 months.

### Statistical analysis

The primary analysis will estimate the mean difference in AWD between the two intervention arms using a linear regression model which will include intervention arm, baseline AWD, and the randomisation stratification variable ‘centre’ as covariates. A repeated measure analysis will be undertaken, including patient as a random effect, to investigate the relationship between AWD and independent baseline variables such as body mass index (BMI) with the model including adjustment for centre and baseline AWD. Multiple imputation will be undertaken for all participants who are missing values or drop out by three months. Causal inference methods will be used to estimate treatment effects that account for compliance with the allocated intervention (NMES, SET, EA). Where necessary, data will be transformed to meet normality assumptions. All statistical tests will be two-tailed with a 5% significance level.

Patient characteristics will be summarised. Summaries of continuous variables will be presented as means and standard deviations if normally distributed, and as medians and inter-quartile ranges for skewed data; categorical variables will be presented as frequencies and percentages. Data on the socioeconomic status of patients will be summarised by centre.

**8.3.1. Subgroup analyses**

Subgroup analysis will investigate the effect of the intervention among subgroups and most importantly the cost effectiveness of the intervention. The subgroups that will be considered are NMES + SET, NMES + EA, SET and EA. We will estimate the treatment effect in the six SET sites vs the six non-SET sites (NMES+SET & SET vs NMES +EA & EA). We will also estimate the treatment effect of NMES in the SET and non-SET sites (NMES+SET vs SET and NMES+EA vs EA).

We will also investigate if NMES + EA has a similar effect as SET alone and if NMES + EA is more cost effective than SET alone when accounting for compliance and using causal inference methods. Also we will aim at determining if NMES + SET is more effective than NMES + EA or SET alone. The subgroup effects will be based on the interaction between subgroup and treatment arm.

All analyses will be on an intention-to-treat basis where all participants will be analysed in the groups to which they were allocated regardless of the treatment they received. Per-protocol analyses will also be performed.

A statistical analysis plan including a detailed description of all the planned analysis will be prepared, finalised and signed off before the database lock. There is no planned interim analysis.

**8.3.2. Mechanistic Evaluation**

The mechanistic evaluation of the interventions will involve haemodynamic measurements of femoral artery blood flow including time averaged mean velocity (TAMV, cm/s) and blood flow (cc/min) by DU and skin microcirculation (blood flux) by LDF.

These are both continuous measurements; haemodynamic measurements via LDF will be taken several times during the trial assessment time points (baseline, 3 months, 6 months, 12 months) DU will be conducted at baseline and months only. The main analysis will involve comparing arterial flow and skin microcirculation at 3 months for the intervention and control group using multi-level regression to take into account the repeated measurements.

A secondary analysis will investigate the effect of compliance and drop out by calculating the complier average causal effect.

**8.3.3. Cost-effectiveness analysis**

The economic analyses will compare local therapy (supervised exercise therapy, SET or exercise advice, EA only) versus the intervention (local therapy + Neuromuscular Electrical Stimulation, NMES) in patients with Intermittent Claudication. The price year will be 2017/2018. The analyses will be performed from the perspective of the NHS. Secondary analyses will consider the societal perspective. As a preliminary step, a literature review will be conducted to identify other economic analyses in these or similar patients. The analyses will be based on a (i) patient level in-trial cost-effectiveness analysis, (ii) a decision model and (iii) budget impact assessment. The in-trial analysis will calculate costs and quality-adjusted life years (QALYs) over the one year time horizon of the trial. Appropriate methods will be used to handle missing data. Costs will be estimated from healthcare resource use in the trial. Unit costs will be taken from the literature and manufacturers’ list prices. QALYs will be measured from EQ-5D-5L collected in the trial adjusting for baseline differences in utility. Subgroup analyses will be carried out by pre-defined groups as described in the statistical analysis plan. The decision model will use the information from the trial and other sources to project costs and QALYs over the lifetime of the patients, by estimating the effect of IC on mortality, quality of life and resource use, and the impact of the intervention on these endpoints. As Intermittent Claudication is a chronic disease, a Markov structure is likely to be appropriate. Costs and QALYs will be discounted. Univariate sensitivity analyses will be used to test the robustness of the model to alternative input parameters. Probabilistic sensitivity analyses will be undertaken using Monte-Carlo simulation. The budget impact assessment will estimate the annual cost to the NHS taking into account projected take-up of the treatment, should it be demonstrated to be cost-effective, and expected cost savings to the NHS. Pseudonymised data will be transferred to the University of Granada in accordance with a data sharing agreement, collaboration agreement, and ICTU SOPs

## REGULATORY, ETHICAL AND LEGAL ISSUES

### Declaration of Helsinki

The investigator will ensure that this study is conducted in full conformity with the *7^th^* revision (2013) of the 1964 Declaration of Helsinki.

### Good Clinical Practice

The study will be conducted in accordance with the guidelines laid down by the International Conference on Harmonisation for Good Clinical Practice (ICH GCP E6 R2 guidelines).

### Independent Ethics Committee Approval

#### Initial Approval

Prior to the shipment of device and the enrolment of subjects, the REC and HRA must provide written approval of the conduct of the study at named sites, the protocol and any amendments, the Subject Information Sheet and Consent Form, any other written information that will be provided to the subjects, any advertisements that will be used and details of any subject compensation.

#### 9.3.2. Approval of Amendments

Amendments to the protocol will only be made with the approval of the Chief

Investigator and will be subject to review and approval by the Sponsor who will decide whether the changes are substantial or non-substantial.

Proposed amendments to the protocol and aforementioned documents must be submitted to the REC for approval as instructed by the Sponsor. Amendments requiring REC approval may be implemented only after a copy of the REC’s approval letter has been obtained.

Amendments that are intended to eliminate an apparent immediate hazard to subjects may be implemented prior to receiving Sponsor or REC and HRA’s approval. However, in this case, approval must be obtained as soon as possible after implementation.

#### 9.3.3 Annual Progress Reports

The REC will be sent annual progress reports in accordance with national requirements.

#### 9.3.4 Annual Safety Reports and End of Trial Notification

The REC will be sent annual safety updates in order to facilitate their continuing review of the study (reference. ICH GCP E6 Section 3.1.4) and will also be informed about the end of the trial, within the required timelines.

### Regulatory Authority Approval

The study will be performed in compliance with UK regulatory requirements. A notice of no objection from the appropriate Regulatory Authority must be obtained prior to the start of the study. In addition, the Regulatory Authority must approve amendments prior to their implementation (as instructed by the Sponsor), receive SUSAR reports and annual safety updates, and be notified of the end of the trial.

### HRA approval

Health Research Authority (HRA) approval will be obtained prior to starting the study. Each participating site will confirm capacity and capability prior to commencing.

The HRA and all participating sites also need to be notified of all protocol amendments to assess whether the amendment affects the institutional approval for each site.

### Non-Compliance and Serious Breaches

All protocol deviations and protocol violations will be reported via the eCRF/CRF and reviewed by the Chief Investigator and reported to the ICTU QA Manager on a monthly basis. Protocol violations will be reported to the Sponsor.

An assessment of whether the protocol deviation/violation constitutes a serious breach will be made.

A serious breach is defined as:

A breach of the conditions and principles of GCP in connection with a trial or the trial protocol, which is likely to affect to a significant degree:

- The safety or physical or mental integrity of the UK trial subjects; or
- The overall scientific value of the trial

The Sponsor will be notified within 24 hours of identifying a likely serious breach. If a decision is made that the incident constitutes a Serious Breach, this will be reported to the MHRA and REC within 7 days of becoming aware of the serious breach.

### Insurance and Indemnity

Imperial College London holds negligent harm and non-negligent harm insurance policies which apply to this study. Imperial College London will act as the main Sponsor for this study. Delegated responsibilities will be assigned to the NHS trusts taking part in this study.

### Trial Registration

The study will be registered on a trial database (ISRCTN and clinicaltrials.gov) in accordance with requirements of the International Committee of Medical Journal Editors (ICMJE) regulations.

### Informed Consent

Consent to enter the study will be sought from each participant only after a full verbal explanation has been given, and an information leaflet offered. The consent will be informed, voluntary and participants will be given an appropriate amount of time to consider participation and to ask questions. There will be no set minimum time to consider the trial as this will be determined on a case by case basis, this is usually 24 hours but could be less if there is agreement from both the researcher and participant that the consent is fully informed.

Signed participant consent will be obtained and participants will be asked to consent for their data to be linked with appropriate databases including Hospital Episode Statistics (HES), and the National Vascular Database as well as for longer term follow-up in the event the trial is extended. A copy of the signed Participant Information Sheet/Informed Consent Form document will be provided to the patient and the original Informed Consent Form should be retained with the source documents

The right of the participant to refuse to participate without giving reasons will be respected, although if the participate is willing a reason for declining will be recorded.

After the participant has entered the trial the clinician remains free to give alternative treatment to that specified in the protocol at any stage if he/she feels it is in the participant’s best interest, but the reasons for doing so should be recorded. In these cases, the participants remain within the study for the purposes of follow-up and data analysis. All participants are free to withdraw at any time from the protocol treatment without giving reasons and without prejudicing further treatment. Participants will be asked to consent to long term follow up to allow for linkage to routine datasets including Hospital Episode Statistics (HES) and the National Vascular Database.

### Contact with General Practitioner

It is the investigator’s responsibility to inform the subject’s General Practitioner (where applicable) by letter that the subject is taking part in the study provided the subject agrees to this, and information to this effect is included in the Subject Information Sheet and Informed Consent. A copy of the letter should be filed in the Investigator Site File.

### Subject Confidentiality

The investigator must ensure that the subject’s confidentiality is maintained. On the CRF or other documents submitted to the Sponsor, or on anonymised safety information provided to the device manufacturer, subjects will be identified by a subject ID number only.

Documents that are held by the coordinating centre (e.g., signed informed consent form) should be kept in a lockable office in a strictly confidential file by the investigator.

The investigator shall permit direct access to subjects’ records and source documents for the purposes of monitoring, auditing, or inspection by the Sponsor, authorised representatives of the Sponsor, NHS and Regulatory Authorities.

Subject’s mobile phone numbers will be entered into a secure database hosted by a company called The 3^rd^ Degree who will send weekly text message reminders. This company is regularly used by the NHS and will keep subjects’ phone numbers confidential. Subjects will only receive reminder messages for the duration of the study or up until the point of withdrawal, loss to follow up or at the patient’s request.

### Data Protection and Patient Confidentiality

The investigator will preserve the confidentiality of all participants taking part in the study, which will be conducted in accordance with the Data Protection Act.

### End of Trial

The end of the trial is defined as last patient last visit.

### Study Documentation and Data Storage

The investigator must retain essential documents until notified by the Sponsor, and for at least ten years after study completion. Subject files and other source data (including copies of protocols, CRFs, original reports of test results, correspondence, records of informed consent, and other documents pertaining to the conduct of the study) must be retained. Documents should be stored in such a way that they can be accessed/data retrieved at a later date. Consideration should be given to security and environmental risks.

No study document will be destroyed without prior written agreement between the Sponsor and the investigator. Should the investigator wish to assign the study records to another party or move them to another location, written agreement must be obtained from the Sponsor.

## DATA MANAGEMENT

### Source Data

Data will be written directly into the CRF and then transcribed into the eCRF. Source documents include original documents related to the trial, to medical treatment and to the history of the participant, and adequate source documentation must be maintained to allow reliable verification and validation of the trial data.

### Language

CRFs will be in English. Generic names for concomitant medications should be recorded in the CRF wherever possible. All written material to be used by subjects must use vocabulary that is clearly understood, and be in the language appropriate for the study site.

### Database

The principal means of data collection from participant visits will be Electronic Data Capture (EDC) in the InForm database system via the internet. Data is entered into the EDC system via site personnel. All source data recorded in the CRF will be signed by the Investigator or his/her appropriate designee. All changes made following the electronic signing will have an electronic audit trail with a signature and date. Specific instructions and further details will be outlined in SOPs and/or manuals.

### Data Collection

Details of procedures for eCRF/CRF completion will be provided in a study manual.

### Archiving

All trial documentation, including that held at participating sites and the trial coordinating centre, will be archived for a minimum of 10 years following the end of the study.

## STUDY MANAGEMENT STRUCTURE

### Trial Steering Committee

In line with current NIHR recommendations a Trial Steering Committee (TSC) will be convened and will include as a minimum an independent Chair, independent clinician, the Chief Investigator and Trial Manager. A lay member will be invited to join the committee.

The role of the TSC is to provide overall supervision of trial conduct and progress. Details of membership, responsibilities and frequency of meetings will be conducted as per the EME research governance guidelines and are defined in a separate TSC Charter. A TSC meeting will be held at the start of the trial prior to patient recruitment, and then annually as a minimum.

### Trial Management Group

A Trial Management Group (TMG) will be convened including the Chief Investigator, co-investigators and key collaborators, trial statistician and trial manager. The TMG will be responsible for day-to-day conduct of the trial and operational issues. Details of membership, responsibilities and frequency of meetings will be defined in separate terms of Reference. A lay person should be included wherever possible. A lay member will be invited to join the group.

TMG meetings will be held monthly throughout the set up and recruitment phase and then at least every other month until trial closure.

### Data Monitoring Committee

In line with current NIHR recommendations a Data Monitoring Committee (DMC) will be convened and will include as a minimum a clinician with experience in the relevant area and expert trial statistician.

The role of the DMC is to monitor patient safety and treatment efficacy data. Details of membership, responsibilities and frequency of meetings will be conducted as per the EME research governance guidelines and are defined in a separate DMC Charter. A DMC meeting will be held prior to first patient first visit, following completion of an internal pilot study and will then be held one month prior to each TSC meeting.

### Early Discontinuation of the Study

There are no formal stopping rules, but safety will be reviewed periodically by the DMC who could recommend early discontinuation of the study.

### Risk Assessment

A study-specific risk assessment will be performed prior to the start of the study to assign a risk category of ‘low’, ‘medium’ or ‘high’ to the trial. Risk assessment will be carried out by the ICTU QA Manager in collaboration with the Study Manager and the result will be used to guide the monitoring plan. The risk assessment will consider all aspects of the study and will be updated as required during the course of the study.

### Monitoring

The study will be monitored periodically by trial monitors to assess the progress of the study, verify adherence to the protocol, ICH GCP E6 guidelines and other national/international requirements and to review the completeness, accuracy and consistency of the data.

Monitoring procedures and requirements will be documented in a Monitoring Plan, developed in accordance with the risk assessment and will follow a risk based approach when determining the level of onsite and remote monitoring.

### Quality Control and Quality Assurance

Quality Control will be performed according to ICTU internal procedures. The study may be audited by a Quality Assurance representative of the Sponsor and/or ICTU. All necessary data and documents will be made available for inspection.

The study may be subject to inspection and audit by regulatory bodies to ensure adherence to GCP and the NHS Research Governance Framework for Health and Social Care (2^nd^ Edition).

### Peer review

The study design was peer reviewed by NIHR funding panel which includes at least 7 different reviewers, including expert clinicians, lay representatives and statisticians.

### Patient and Public Involvement

Participation, involvement and engagement are key principles to running an effective study in order to obtain the right balance between academic development and user satisfaction. PPI will be incorporated to the following areas of the research project:

- **Design of the research**: An experienced patient representative is a co-applicant of this study and has played a key advisory role in providing an understandable plain English summary as detailed in the outline submission and, as part of a number of patient representatives, provided a key patient perspective on achieving our project aims, having been a participant in a similar trial.
- **Management of the research**: This patient co-applicant has expressed a keenness to continue on-going active involvement including from an advisory perspective at various time points in this study and will be invited to be an active member of the TSC and be involved in TMG discussions.
- **Undertaking the research:** Activities will include the development of patient participation information sheets and associated documents in addition to vital end-user input throughout the study period.
- **Analysis of results:** The patient representative will also be invited to take part in the analysis discussions.
- **Dissemination of findings:** The patient representative will also be invited to provide perspective during dissemination of study findings including how best to communicate these to study participants and other patient groups.

### Publication and Dissemination policy

This research will be beneficial for health care professionals, patients, NHS policy makers and commissioners. Dissemination of the study findings will be undertaken on an ongoing basis, appropriate for these groups and publications will be written in accordance with the Consort guidelines.

Briefing papers will summarise the key findings relevant to this study and executive summaries of main reports will be beneficial for NHS policy makers. The study protocol and results will be written up and submitted for publication in a high impact factor medical journal. In order to reach a wider audience, open access publication has been factored into the costings of this trial.

Regular presentation of study findings will be made to nurses, doctors and allied health professionals through local, regional, national and international meetings of the research network. Study findings will also be disseminated through important national professional organisations including the Vascular Society of Great Britain and Ireland, The Royal Society of Medicine and The Circulation Foundation. Study findings will also be made available via online media through a study website and social media including Twitter, Facebook and LinkedIn. The trial management group will draft reports and each centre involved in the study will be provided with copies of manuscripts for review of all lead investigators and collaborators prior to publication.

Workshops will be held with key stakeholders including patient and public groups, allied health professionals and policy makers to discuss the study findings and methods of implementation in more detail.

Information concerning the study, patent applications, processes, scientific data or other pertinent information is confidential and remains the property of the Sponsor. The investigator may use this information for the purposes of the study only.

It is understood by the investigator that the manufacturer may use information developed in this clinical study in connection with the development of the device and, therefore, may disclose it as required to other clinical investigators and to Regulatory Authorities. In order to allow the use of the information derived from this clinical study, the investigator understands that he/she has an obligation to provide complete test results and all data developed during this study to the Sponsor.

Verbal or written discussion of results prior to study completion and full reporting should only be undertaken with written consent from the Sponsor. Therefore, all information obtained as a result of the study will be regarded as CONFIDENTIAL, at least until appropriate analysis and review by the investigator(s) are completed.

Permission from the Executive/Writing Committee is necessary prior to disclosing any information relative to this study outside of the Trial Steering. Any request by site investigators or other collaborators to access the study dataset must be formally reviewed by the TSC.

The results may be published or presented by the investigator(s), but the Funder will be given the opportunity to review and comment on any such results for up to 1 month before any presentations or publications are produced.

A Clinical Study Report summarising the study results will be prepared and submitted to the REC and MHRA within a year of the end of study. The results will also be submitted to the EudraCT results database in accordance with regulatory requirements.

## REFERENCES

1. Fowkes FG, Rudan D, Rudan I, Aboyans V, Denenberg JO, McDermott MM, et al. Comparison of global estimates of prevalence and risk factors for peripheral artery disease in 2000 and 2010: a systematic review and analysis. Lancet.382(9901):1329-40.
2. Norgren L, Hiatt WR, Dormandy JA, Nehler MR, Harris KA, Fowkes FGR. Inter-Society Consensus for the Management of Peripheral Arterial Disease (TASC II). Journal of Vascular Surgery. 2007;45(1

SUPPL.):S5-S67.

1. Fowkes FG, Housley E, Cawood EH, Macintyre CC, Ruckley CV, Prescott RJ. Edinburgh Artery Study: prevalence of asymptomatic and symptomatic peripheral arterial disease in the general population. International Journal of Epidemiology.20(2):384-92.
2. National Institute of Health and Care Excellence (NICE). Lower Limb Peripheral Arterial Disease: Diagnosis and Management. NICE Clinical Guideline 147. 2012;gudance.nice.org.uk/cg147:1-30.
3. Bendermacher BL, Willigendael EM, Teijink JA, Prins MH. Supervised exercise therapy versus non-supervised exercise therapy for intermittent claudication. Cochrane database of systematic reviews (Online). 2006(2):CD005263.
4. Fokkenrood HJ, Bendermacher BL, Lauret GJ, Willigendael EM, Prins MH, Teijink JA. Supervised exercise therapy versus non-supervised exercise therapy for intermittent claudication. The Cochrane database of systematic reviews. 2013;8:Cd005263.
5. Shalhoub J, Hamish M, Davies AH. Supervised exercise for intermittent claudication - an under-utilised tool. Annals of the Royal College of Surgeons of England. 2009;91(6):473-6.
6. Babber A DSL, Ge Y, Davies A. SARS abstracts: Availability of supervised exercise programmes for patients with peripheral arterial disease. 0159. British Journal of Surgery. 2015;102(s5):46.
7. Bermingham SL, Sparrow K, Mullis R, Fox M, Shearman C, Bradbury A, et al. The cost-effectiveness of supervised exercise for the treatment of intermittent claudication. European journal of vascular and endovascular surgery : the official journal of the European Society for Vascular Surgery. 2013;46(6):707-14.
8. Babber A RR, Williams K, Davies AH. Neuromuscular Electrical Stimulation in the Management of Peripheral Arterial Disease: A 'Stimulating' Prospect. Journal of Vascular Surgery. 2016;63(6):16S
9. Williams KJ, Babber A, Ravikumar R, Davies AH. Non-Invasive Management of Peripheral Arterial Disease Medicine and Biology - Advances in Experimental Medicine and Biology. 2016;1:387-406.
10. Delis KT, Nicolaides AN. Effect of intermittent pneumatic compression of foot and calf on walking distance, hemodynamics, and quality of life in patients with arterial claudication: a prospective randomized controlled study with 1-year follow-up. Annals of Surgery.241(3):431- 41.
11. Bemmelen PSV MM, Faught WE, Ashraf Mansour M, Barkmeier D, Hodgson KJ, Ramsey DE, Summer DS. Augmentation of blood flow in limbs with occlusive arterial disease by intermittent calf compression. Journal of Vascular Surgery. 1994;19(6):1052-8.
12. Williams KJ MH, Davies AH. Haemodynamic changes with the use of compression. Phlebology / Venous Forum of the Royal Society of Medicine
13. Gardner AW, Skinner JS, Cantwell BW, Smith LK. Progressive vs Exerc. 1991;23(4):402-8.
14. Varatharajan L, Williams K, Moore H, Davies AH. The effect of footplate neuromuscular electrical stimulation on venous and arterial haemodynamics. Phlebology / Venous Forum of the Royal Society of Medicine. 2015;30(9):648-50.
15. Greenhalgh RM, Belch JJ, Brown LC, Gaines PA, Gao L, Reise JA, et al. The adjuvant benefit of angioplasty in patients with mild to moderate intermittent claudication (MIMIC) managed by supervised exercise, smoking cessation advice and best medical therapy: results from two randomised trials for stenotic femoropopliteal and aortoiliac arterial disease. European Journal of Vascular & Endovascular Surgery.36(6):680-8.
16. Cheetham DR, Burgess L, Ellis M, Williams A, Greenhalgh RM, Davies AH. Does supervised exercise offer adjuvant benefit over exercise advice alone for the treatment of intermittent claudication? A randomised trial. European Journal of Vascular and Endovascular Surgery. 2004;27(1):17-23.
17. Perkins JMT, Collin J, Creasy TS, Fletcher EWL, Morris PJ. Exercise training versus angioplasty for stable claudication. Long and medium term results of a prospective, randomised trial. European Journal of Vascular and Endovascular Surgery. 1996;11(4):409-13.
18. Mazari FAK, Khan JA, Carradice D, Samuel N, Abdul Rahman MNA, Gulati S, et al. Randomized clinical trial of percutaneous transluminal angioplasty, supervised exercise and combined treatment for intermittent claudication due to femoropopliteal arterial disease. British Journal of Surgery. 2012;99(1):39-48.
19. Watson T. Electrotherapy: Evidence-Based Practice. 12th Edition edn.
20. Corley GJ, Breen PP, Grace PA, G OL. The effect of surface neuromuscular electrical stimulation and compression hosiery applied to the lower limb, on the comfort and blood flow of healthy subjects. Conf Proc IEEE Eng Med Biol Soc 2008;2008:703-6. <http://dx.doi.org/10.1109/IEMBS.2008.4649249>

# SIGNATURE PAGE 1 (Chief Investigator)

The signature below constitutes approval of this protocol by the signatory and provides the necessary assurances that this study will be conducted according to all stipulations of the protocol including all statements regarding confidentiality.

**Study Title:** A Multicentre Randomised Controlled Study: Does **N**euromuscular **E**lectrical **S**timulation Improve the Absolute Walking Distance in Patients with **I**ntermittent **C**laudication (NESIC) compared to best available treatment?

**Protocol Number:** **6.0**

Signed: ___________________________________________

Alun H Davies

Professor

Date: _____________________

# SIGNATURE PAGE 2 (SPONSOR)

The signatures below constitute approval of this protocol by the signatory.

**Study Title:** A Multicentre Randomised Controlled Study: Does **N**euromuscular **E**lectrical **S**timulation Improve the Absolute Walking Distance in Patients with **I**ntermittent **C**laudication (NESIC) compared to best available treatment?

**Protocol Number:** **6.0**

Signed: ___________________________________________

Becky Ward

Ms

Imperial College London

Date: _____________________

# SIGNATURE PAGE 3 (STATISTICIAN)

The signatures below constitute approval of this protocol by the signatory.

**Study Title:** A Multicentre Randomised Controlled Study: Does **N**euromuscular **E**lectrical **S**timulation Improve the Absolute Walking Distance in Patients with **I**ntermittent **C**laudication (NESIC) compared to best available treatment?

**Protocol Number:** **6.0**

Signed: ___________________________________________

Francesca Fiorentino

Dr.

Imperial College London

Date: _____________________

# SIGNATURE PAGE 4 (principal INVESTIGATOR)

The signature of the below constitutes agreement of this protocol by the signatory and provides the necessary assurance that this study will be conducted at his/her investigational site according to all stipulations of the protocol including all statements regarding confidentiality.

**Study Title:** A Multicentre Randomised Controlled Study: Does **N**euromuscular **E**lectrical **S**timulation Improve the Absolute Walking Distance in Patients with **I**ntermittent **C**laudication (NESIC) compared to best available treatment?

**Protocol Number:** **6.0**

Address of Institution: ____________________________________________

____________________________________________

Signed: ____________________________________________

Print Name and Title: ____________________________________________

Date: ____________________________________________

**Supplementary Appendixes**

**eAppendix 1. List of Investigators**

The NESIC team would like to thank the NHS trusts and participating principal investigators and their colleagues for recruiting and monitoring trial participants. These include (in alphabetical order of participating hospitals followed by the local principal investigators and their colleagues’): Addenbrooke’s Hospital, Cambridge – M.S. Gohel, A. Pentelow, P. Shipley-Cribb, R. Elliot, N. Nacorda, R. Ward, D. Read; Charing Cross Hospital, London – A.H. Davies, J. Shalhoub, T. Lane, L. Bolton, T. V. Le-Magowan, L. Burgess, B. Jones, N. Strevens, A. M. Malagoni, S. Tavares, A. Henry, C. Connelly, J. Smee, R. Toledano, J. Nunag, L. Tarusan, N. Yasmin, C. Carr; Dorset County Hospital – J. Metcalfe, B. Page, S. Williams, D. Hill, G. Belt, A. Rees, S. Palmer, S. Horton, D. Lovelock; Freeman Hospital, Newcastle – G. Stansby, N. Parr, M. Catterson, E. Scott, L. Wales, J. McCaslin, M. Clarke, S. Kirkup, D. Amis, A. Robinson, A. Phillipson, S. Covill, V. Wealleans, E. Fairbairn; Hull Royal Infirmary – I. Chetter, A. Harwood, J. Long, J. Totty, A. Mohamed, T. Wallace, J. Hatfield, P. Cai, S. Pymer, J. Palmer, A. Firth. T. Roe, S. Ibeggazene, L. Andrews; Musgrove Park Hospital, Taunton – J. Coulston, A. Stewart, K. Roberts, J. Rewbury, S. Mitchell, H. Mills, L. Vickery, C. Adams, S. Shakya, R. Hadley, L. Timewell, C. Williams, J. Kanapathipillai, J. Hutter, F. Goodchild, N. Greig, J. Blackall, K. O’Callaghan, J. Lucas; Queen's Medical Centre, Nottingham – B. Braithwaite, R. Simpson, R. Hadley; Royal Bournemouth General Hospital – D. Rittoo, C. Thomson, L. Vamplew, M. Letts, T. Webb, E. Howe, A. Fraine, J. Kelly, F. Beecham; Southampton General Hospital – N. Pal, M. Hulse, P. Patel, I. Nordon, S. Smith, F. Smith, H. Yates, C. Boxall, J. Harvey, S. Hammond; Southmead Hospital, Bristol – R. Hinchliffe, H. Cheshire, K. Harding, S. McIntosh, L. Poole, P. Brock; St George's Hospital, London – P. Holt, N. Sachsinger, R. Ingham, J. Budge, J. Pang, P. Ribeiro.

**eAppendix 2. Trial Committees**

We would also like to thank members of our two oversight committees; Trial Steering Committee: Professor Andrew Bradbury (Chair); Professor Jonathan Beard (Consultant Vascular Surgeon); Dr Louise Brown (Medical Statistician) and Mr Sydney Chapple (lay member) who provided invaluable input and advice as the independent lay member over the course of the study; Data Monitoring Committee Professor Julie Brittenden (Chair, Professor of Vascular Surgery); Mr Chung Sim Lim (Consultant Vascular Surgeon); Dr Stephen Gerry (Medical Statistician) for their support and guidance.

**eAppendix 3. List of inclusion and exclusion criteria**

Inclusion Criteria:

- Capacity to provide informed consent
- Aged 18 or above
- Positive Edinburgh Claudication Questionnaire
- ABPI <0.9 OR positive stress test (fall in ankle pressure >30mmHg, 40 secs post 1 min treadmill at 10% gradient, 4 km/h)

Exclusion Criteria:

- Severe IC requiring invasive intervention as determined by the treating clinician
- Critical limb Ischaemia as defined by the European Consensus Document
- Co-morbid disease prohibiting walking on a treadmill or taking part in supervised exercise therapy
- Able to walk for longer than 15 minutes on the study treadmill assessment
- Have attended supervised exercise therapy classes in the previous 6 months
- Popliteal entrapment syndrome
- Commenced vascular symptom specific medication in previous 6 months e.g. naftidrofuryl oxalate, cilostazol
- Pregnancy
- Any implanted electronic, cardiac or defibrillator device
- Acute deep vein thrombosis
- Broken or bleeding skin including leg ulceration
- Peripheral neuropathy
- Recent lower limb injury or lower back pain
- Already using a neuromuscular electrical stimulation device

**eAppendix 4. End of study**

Participants randomised to receive NMES were able to keep the Revitive™ IX device and participants randomised to local standard care only were offered a device at their 12-month visit.

**eAppendix** **5. EuroQol-5D-5L**


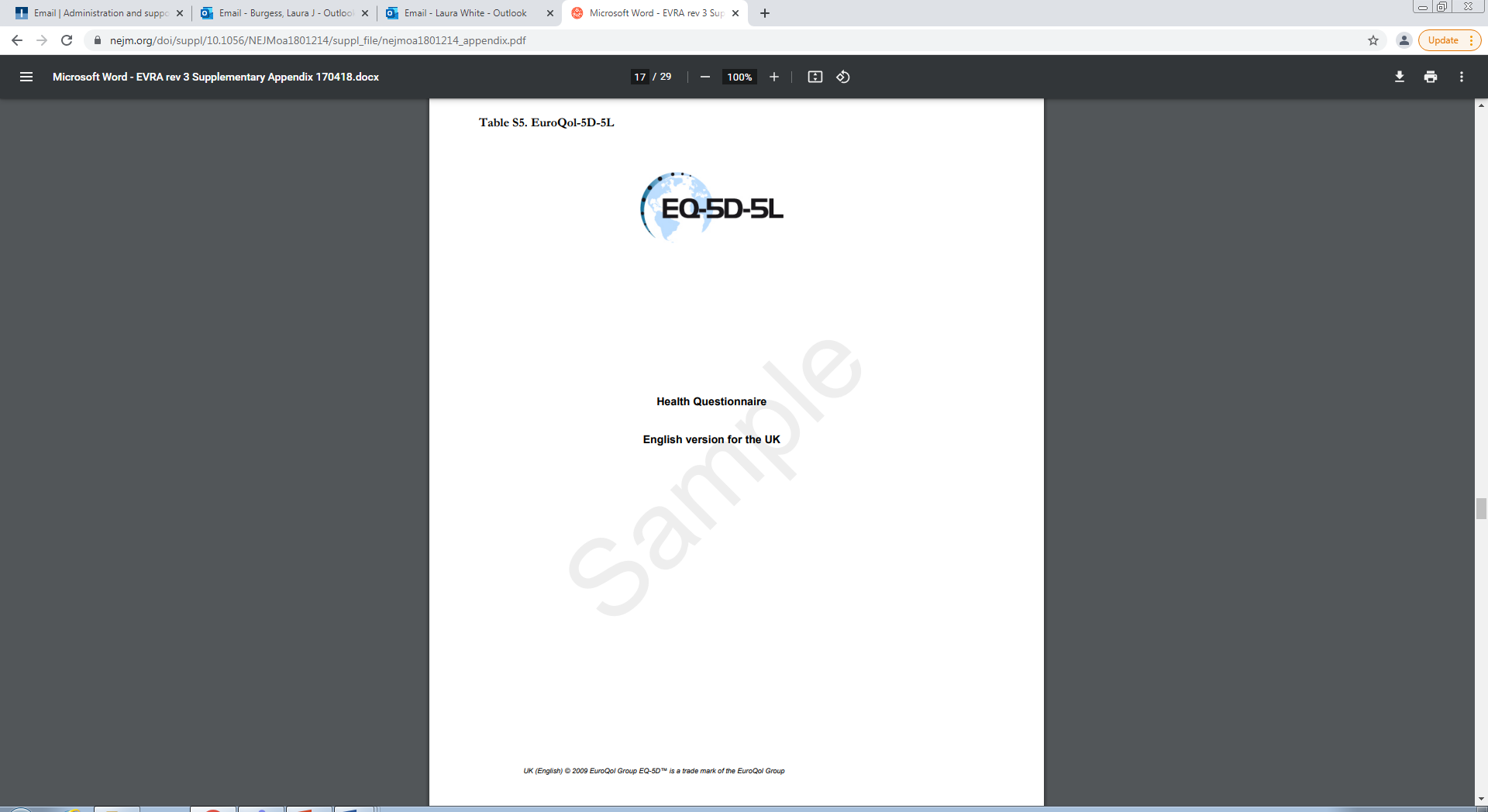


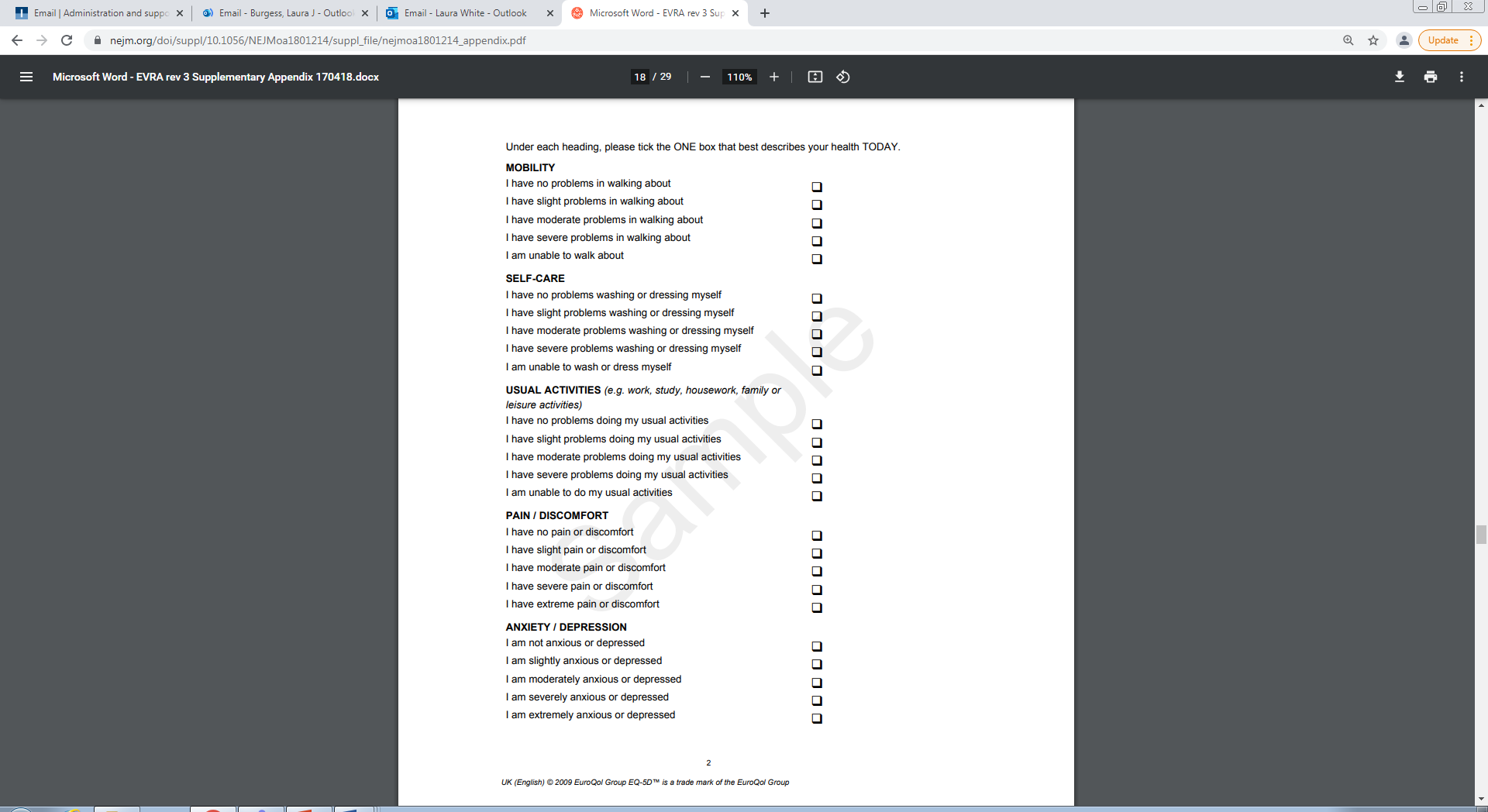


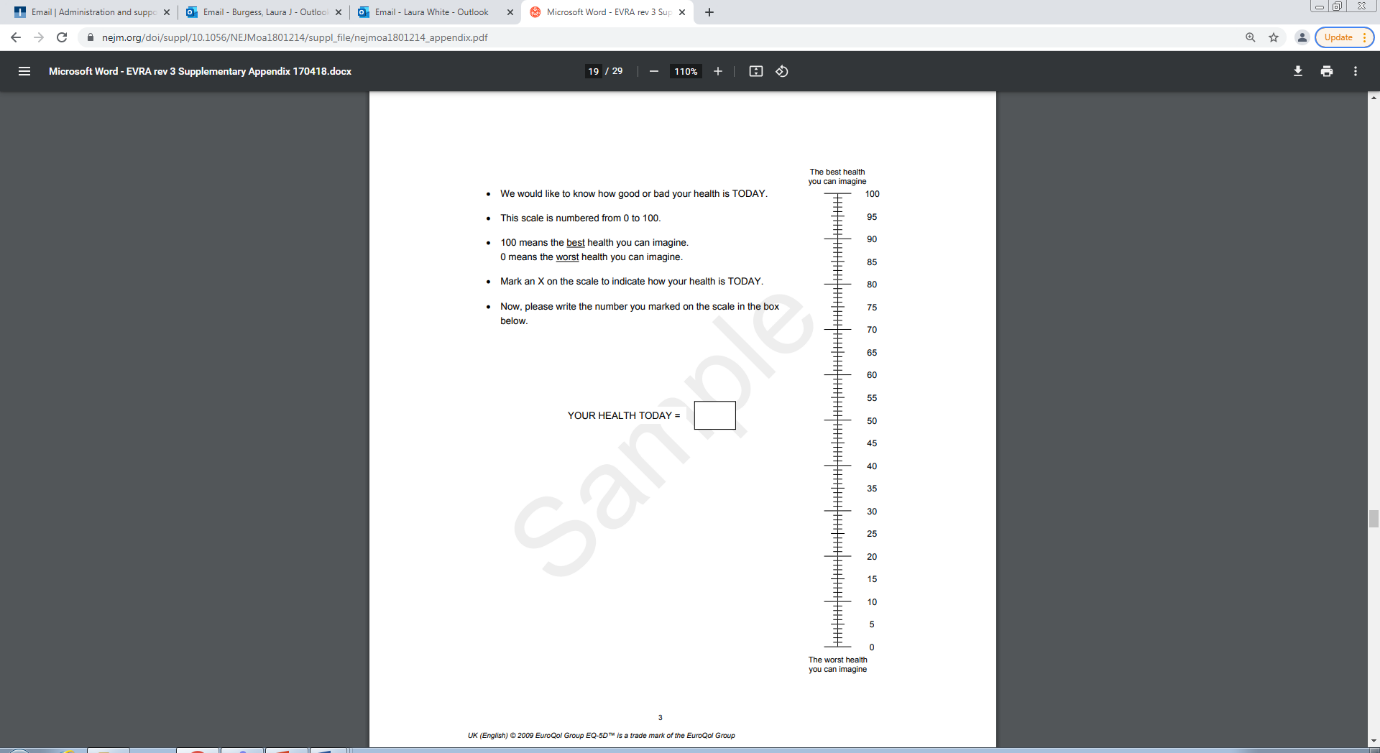


**eAppendix 6. Intermittent Claudication Questionnaire**

Intermittent Claudication Questionnaire (ICQ)

**The questions below ask about the problems of pains, cramps, and numbness or discomfort that patients with arterial disease get in their calves, thighs or buttocks when walking. The term “leg pains” has been used to describe all of these problems. Please answer every question with a tick. If you are unsure about how to answer a question, please give the best answer you can. When answering each question think about any pains, cramps, numbness or discomfort that you may get in your calves, thighs or buttocks when walking. Tick only one box per question.**

1. During the past 2 weeks, how severe were your leg pains?

|  |  |  | | |  |  |  | |  |  | |  |  | |  |  | |  |  |
| --- | --- | --- | --- | --- | --- | --- | --- | --- | --- | --- | --- | --- | --- | --- | --- | --- | --- | --- | --- |
| None, I had no leg pain | | |  | Very mild | | | | Mild | | | Moderate | | | Severe | | | Very severe | | |

The following questions ask about activities you might do during a typical day. Do your leg pains limit you in these activities? If so, how much?

|  | | **Totally**  **limited** | | | **Very**  **limited** | | | **Moderately**  **limited** | | | **A little**  **limited** | | | **Not limited**  **at all** | | |  |
| --- | --- | --- | --- | --- | --- | --- | --- | --- | --- | --- | --- | --- | --- | --- | --- | --- | --- |
| 2. | Crossing the |  |  |  |  |  |  |  |  |  |  |  |  |  |  |  | |
|  | Road |  |  |  |  |  |  |  |  |  |  |  |  |  |  |  | |
|  |  |  |  |  |  |  |  |  |  |  |  |  |  |  |  |  | |
| 3. | Using the bus |  |  |  |  |  |  |  |  |  |  |  |  |  |  |  | |
|  | Train or tube |  |  |  |  |  |  |  |  |  |  |  |  |  |  |  | |
|  |  |  |  |  |  |  |  |  |  |  |  |  |  |  |  |  | |
| 4. | Climbing |  |  |  |  |  |  |  |  |  |  |  |  |  |  |  | |
|  | Several flights of |  |  |  |  |  |  |  |  |  |  |  |  |  |  |  | |
|  | Stairs |  |  |  |  |  |  |  |  |  |  |  |  |  |  |  | |
|  |  |  |  |  |  |  |  |  |  |  |  |  |  |  |  |  | |
| 5. | Climbing one |  |  |  |  |  |  |  |  |  |  |  |  |  |  |  | |
|  | Flight of stairs |  |  |  |  |  |  |  |  |  |  |  |  |  |  |  | |
|  |  |  |  |  |  |  |  |  |  |  |  |  |  |  |  |  | |
| 6. | Walking more |  |  |  |  |  |  |  |  |  |  |  |  |  |  |  | |
|  | Than a mile |  |  |  |  |  |  |  |  |  |  |  |  |  |  |  | |
|  |  |  |  |  |  |  |  |  |  |  |  |  |  |  |  |  | |
| 7.  yards | Walking 100 |  |  |  |  |  |  |  |  |  |  |  |  |  |  |  | |
|  |  |  |  |  |  |  |  |  |  |  |  |  |  |  |  |  | |
|  |  |  |  |  |  |  |  |  |  |  |  |  |  |  |  |  | |
| 8. | Going out of the |  |  |  |  |  |  |  |  |  |  |  |  |  |  |  | |
|  | the house |  |  |  |  |  |  |  |  |  |  |  |  |  |  |  | |

9. During the past 2 weeks, how often have you had to stop walking and rest because of the pains in your leg?

|  |  |  | |  |  | |  |  | |  |  | |  |  |
| --- | --- | --- | --- | --- | --- | --- | --- | --- | --- | --- | --- | --- | --- | --- |
| More than 3 times a day | | | 2 to 3 times a day | | | Once a day | | | Less than once a week | | | Not at all | | |

10. During the past 2 weeks, how much of the time have you spent thinking about your leg pains?

|  |  |  | |  |  | |  |  | |  |  | |  |  |
| --- | --- | --- | --- | --- | --- | --- | --- | --- | --- | --- | --- | --- | --- | --- |
| All of the time | | | Most of the time | | | Some of the time | | | A little of the time | | | None of the time | | |

11. During the past 2 weeks, how much of the time have you felt downhearted and low because of your leg pains?

|  |  |  | |  |  | |  |  | |  |  | |  |  |
| --- | --- | --- | --- | --- | --- | --- | --- | --- | --- | --- | --- | --- | --- | --- |
| All of the time | | | Most of the time | | | Some of the time | | | A little of the time | | | None of the time | | |

12. During the past 2 weeks, how much of the time have you been worried that your leg pains will get worse?

|  |  |  | |  |  | |  |  | |  |  | |  |  |
| --- | --- | --- | --- | --- | --- | --- | --- | --- | --- | --- | --- | --- | --- | --- |
| All of the time | | | Most of the time | | | Some of the time | | | A little of the time | | | None of the time | | |

13. During the past 2 weeks, how much did your leg pains interfere with your normal work (including work both outside the home and housework)?

|  |  |  | |  |  | |  |  | |  |  | |  |  |
| --- | --- | --- | --- | --- | --- | --- | --- | --- | --- | --- | --- | --- | --- | --- |
| Not at all | | | A little bit | | | Moderately | | | Quite a bit | | | Extremely | | |

14. During the past 2 weeks, how much did your leg pains interfere with your hobbies or pastimes?

|  |  |  | |  |  | |  |  | |  |  | |  |  |
| --- | --- | --- | --- | --- | --- | --- | --- | --- | --- | --- | --- | --- | --- | --- |
| Not at all | | | A little bit | | | Moderately | | | Quite a bit | | | Extremely | | |

15. During the past 2 weeks, how much of the time have your leg pains interfered with social activities (like visiting with friends and relatives, etc.)?

|  |  |  | |  |  | |  |  | |  |  | |  |  |
| --- | --- | --- | --- | --- | --- | --- | --- | --- | --- | --- | --- | --- | --- | --- |
| All of the time | | | Most of the time | | | Some of the time | | | A little of the time | | | None of the time | | |

16. During the past 2 weeks, how much of the time have your leg pains interfered with doing errands (like shopping, going to the post office or bank, etc.)?

|  |  |  | |  |  | |  |  | |  |  | |  |  |
| --- | --- | --- | --- | --- | --- | --- | --- | --- | --- | --- | --- | --- | --- | --- |
| All of the time | | | Most of the time | | | Some of the time | | | A little of the time | | | None of the time | | |

**eAppendix 7. Impact of COVID-19**

Due to the COVID-19 crisis, a substantial amendment was submitted to REC in April 2020 to allow all follow-up visits to take place remotely (i.e., over the telephone completely or in combination with postal questionnaires) in the event that the participant is unable to attend in clinic or the site is unable to accommodate the on-site visit. Missed (physical) assessments as a result of a remote visit were re-scheduled at a later date as a separate on-site visit, where possible. If an on-site visit was re-scheduled at a later date, all QoL questionnaires that were completed remotely were repeated at the on-site visit for internal consistency of each participant.

**eAppendix 8. Patient and public involvement (PPI)**

The NESIC collaborators developed a PPI partnership, engaging the public as early as possible and throughout the cycle of the research project to maximize the benefit of the public/patient perspective. During the set-up phase of the trial, patients reviewed all participant-facing documents and assisted with trial design to ensure that the research question and outcomes were relevant to those with the lived experience of the condition. A PPI member was approached to join the trial and agreed to act as a lay patient representative, joining the TSC. Once the trial has been published, participants will be informed of the results of the study through a newsletter suitable for a non-specialist audience.

**eAppendix 9. CONSORT checklist**


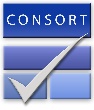
CONSORT 2010 checklist of information to include when reporting a randomised trial*

| Section/Topic | Item No | Checklist item | Reported on page No |
| --- | --- | --- | --- |
| Title and abstract | | | |
|  | 1a | Identification as a randomised trial in the title | 1 (Title Page section) |
|  | 1b | Structured summary of trial design, methods, results, and conclusions (for specific guidance see CONSORT for abstracts) | 1 |
| Introduction | | | |
| Background and objectives | 2a | Scientific background and explanation of rationale | 3-4 |
|  | 2b | Specific objectives or hypotheses | 4 |
| Methods | | | |
| Trial design | 3a | Description of trial design (such as parallel, factorial) including allocation ratio | 5 |
|  | 3b | Important changes to methods after trial commencement (such as eligibility criteria), with reasons | N/A |
| Participants | 4a | Eligibility criteria for participants | 5 |
|  | 4b | Settings and locations where the data were collected | 5 |
| Interventions | 5 | The interventions for each group with sufficient details to allow replication, including how and when they were actually administered | 6-7 |
| Outcomes | 6a | Completely defined pre-specified primary and secondary outcome measures, including how and when they were assessed | 7-8 |
|  | 6b | Any changes to trial outcomes after the trial commenced, with reasons | N/A |
| Sample size | 7a | How sample size was determined | 8 |
|  | 7b | When applicable, explanation of any interim analyses and stopping guidelines | N/A |
| Randomisation: |  |  |  |
| Sequence generation | 8a | Method used to generate the random allocation sequence | 5-6 |
|  | 8b | Type of randomisation; details of any restriction (such as blocking and block size) | 5-6 |
| Allocation concealment mechanism | 9 | Mechanism used to implement the random allocation sequence (such as sequentially numbered containers), describing any steps taken to conceal the sequence until interventions were assigned | 5-6 |
| Implementation | 10 | Who generated the random allocation sequence, who enrolled participants, and who assigned participants to interventions | 5-6 |
| Blinding | 11a | If done, who was blinded after assignment to interventions (for example, participants, care providers, those assessing outcomes) and how | N/A |
|  | 11b | If relevant, description of the similarity of interventions | N/A |
| Statistical methods | 12a | Statistical methods used to compare groups for primary and secondary outcomes | 8-9 |
|  | 12b | Methods for additional analyses, such as subgroup analyses and adjusted analyses | 8-9 |
| Results | | | |
| Participant flow (a diagram is strongly recommended) | 13a | For each group, the numbers of participants who were randomly assigned, received intended treatment, and were analysed for the primary outcome | 10 (in separate uploaded figure) |
|  | 13b | For each group, losses and exclusions after randomisation, together with reasons | 10 (in separate uploaded figure) |
| Recruitment | 14a | Dates defining the periods of recruitment and follow-up | 10 |
|  | 14b | Why the trial ended or was stopped | 10 |
| Baseline data | 15 | A table showing baseline demographic and clinical characteristics for each group | 11 |
| Numbers analysed | 16 | For each group, number of participants (denominator) included in each analysis and whether the analysis was by original assigned groups | 12-19 |
| Outcomes and estimation | 17a | For each primary and secondary outcome, results for each group, and the estimated effect size and its precision (such as 95% confidence interval) | 12-19 |
|  | 17b | For binary outcomes, presentation of both absolute and relative effect sizes is recommended | N/A |
| Ancillary analyses | 18 | Results of any other analyses performed, including subgroup analyses and adjusted analyses, distinguishing pre-specified from exploratory | 16-19 |
| Harms | 19 | All important harms or unintended effects in each group (for specific guidance see CONSORT for harms) | 16 |
| Discussion | | | |
| Limitations | 20 | Trial limitations, addressing sources of potential bias, imprecision, and, if relevant, multiplicity of analyses | 20-21 |
| Generalisability | 21 | Generalisability (external validity, applicability) of the trial findings | 20-23 |
| Interpretation | 22 | Interpretation consistent with results, balancing benefits and harms, and considering other relevant evidence | 20-23 |
| Other information | | |  |
| Registration | 23 | Registration number and name of trial registry | 2 |
| Protocol | 24 | Where the full trial protocol can be accessed, if available | 5 |
| Funding | 25 | Sources of funding and other support (such as supply of drugs), role of funders | 2 (Title Page section) |

*We strongly recommend reading this statement in conjunction with the CONSORT 2010 Explanation and Elaboration for important clarifications on all the items. If relevant, we also recommend reading CONSORT extensions for cluster randomised trials, non-inferiority and equivalence trials, non-pharmacological treatments, herbal interventions, and pragmatic trials. Additional extensions are forthcoming: for those and for up to date references relevant to this checklist, see [www.consort-statement.org](http://www.consort-statement.org).

**eTable 1. NESIC study: recruiting centres**

| **NESIC Site** | **Participants recruited** |
| --- | --- |
| Imperial College Healthcare NHS Trust | 30 |
| Cambridge University Hospitals NHS Foundation Trust | 15 |
| North Bristol NHS Trust | 13 |
| Newcastle Upon Tyne Hospitals NHS Foundation Trust | 29 |
| Hull and East Yorkshire Hospitals NHS Foundation Trust | 42 |
| Somerset NHS Foundation Trust  *(formerly Taunton and Somerset NHS Foundation Trust)* | 24 |
| University Hospital Southampton NHS Foundation Trust | 4 |
| Nottingham University Hospitals NHS Trust | 10 |
| Dorset County Hospital NHS Foundation Trust | 16 |
| St George’s University Hospitals NHS Foundation Trust | 10 |
| The Royal Bournemouth & Christchurch Hospitals NHS Foundation Trust | 7 |

**eTable 2. Summary of treatment allocation**

| SET Center Treatment | Number of subjects | Treatment |
| --- | --- | --- |
| Control 1 | 55 | BMT + SET |
| Treatment 1 | 53 | BMT + SET + NMES |
| Total number of subjects SET center | 108 |  |

| \| Non-SET Center Treatment \| Number of subjects \| Treatment \| \| --- \| --- \| --- \| \| Control 2 \| 47 \| BMT \| \| Treatment 2 \| 45 \| BMT + NMES \| \| Total number of subjects non SET center \| 92 \|  \| Total number of trial subjects \| 200 \| \| --- \| --- \| |
| --- | --- | --- | --- | --- | --- | --- | --- | --- | --- | --- | --- | --- | --- |
|  |

**eTable 3. Supervised Exercise Therapy (SET) localised programs**

|  | **Sessions per week** | **Number of months** | **Total number of sessions** |
| --- | --- | --- | --- |
| Imperial College Healthcare NHS Trust | 1 | 6 | 24 |
| North Bristol NHS Trust | 2 | 3 | 24 |
| Hull and East Yorkshire Hospitals NHS Foundation Trust | 3 | 3 | 36 |
| University Hospital Southampton NHS Foundation Trust | 1 | 2 | 8 |
| Dorset County Hospital NHS Foundation Trust | 1 | 2 | 8 |
| The Royal Bournemouth & Christchurch Hospitals NHS Foundation Trust | 1 | 3 | 12 |

**eTable 4. Summary of quality of life tools using in NESIC study**

| **Questionnaire** | **Type of assessment** | **Range of scores** | **Comments** |
| --- | --- | --- | --- |
| EuroQol – 5 Dimension (EQ-5D-5L)[1] | Patient reported generic quality of life | 0 – 100 (health scale)  0 – 1 (health index) | Consists of a health scale and health index, with higher scores indicating better health |
| Short-Form 36 (SF-36) [2] | Patient reported generic quality of life | 0 – 100 (for each domain) | Eight scores covering different domains of health, with higher scores indicating better health |
| Intermittent Claudication Questionnaire (ICQ) [3] | Patient reported disease specific quality of life | 0 – 100 (for each domain) | 5-point adjectival scale with 16 items, with higher scores indicating better health |

**eTable 5. Additional baseline characteristics of the trial participants***

| Characteristic | Treatment: NMES + BMT and NMES+BMT+SET  N=(92) | Control: BMT and BMT+SET  N=(98) |
| --- | --- | --- |
| Race — no. (%) |  |  |
| White | 87 (94.6) | 90 (91.8) |
| Asian | 3 ( 3.3) | 3 (3.1) |
| Black | 1 ( 1.1) | 3 (3.1) |
| Other | 1 ( 1.1) | 2 (2.0) |
| Medical history — no. (%) |  |  |
| Hypertension | 65 (70.7) | 63 (64.3) |
| Stroke | 9 (9.8) | 9 (9.2) |
| Myocardial infarction | 15 (16.3) | 17 (17.3) |
| Hypercholesterolemia | 68 (73.9) | 64 (65.3) |
| Angina | 10 (10.9) | 13 (13.3) |
| Diabetes | 21 (22.8) | 26 (26.5) |
| Bypass revascularisation | 5 (5.4) | 12 (12.2) |
| Angio revascularisation | 14 (15.2) | 25 (25.5) |
| Medication — no. (%) |  |  |
| Antiplatelets | 75 (81.5) | 79 (80.6) |
| Glycoprotein IIb IIIa antagonists | 92 (100.0) | 98 (100.0) |
| Statin | 80 (87.0) | 81 (82.7) |
| Anticoagulant | 10 (10.9) | 15 (15.3) |
| Antihypertensives | 66 (71.7) | 65 (66.3) |

* Plus–minus values are means ±SD. No significant differences were identified between the treatment groups in any baseline variable. Percentages may not total 100 because of rounding.

**eTable 5b Additional baseline characteristics of those who were analysed***

| **Characteristic** | **Treatment: NMES + BMT and NMES+BMT+SET** | **Control: BMT and BMT+SET** |
| --- | --- | --- |
|  | **N=80** | **N=80** |
| **Age (mean±SD)** | 68.59±8.63 years | 67.34±9.48 years |
| **Body mass index (mean±SD)** | 28.23±4.96 kg/m2 | 28.98±6.84 kg/m2 |
| **Sex** |  |  |
| Female | 18 (22.5%) | 25 (31.3%) |
| Male | 62 (77.5%) | 55 (68.8%) |
| **Smoking status** |  |  |
| Current | 18 (22.5%) | 27 (33.8%) |
| Former | 57 (71.3%) | 48 (60.0%) |
| Never | 5 (6.3%) | 5 (6.3%) |
| **ABPI**§ **(mean±SD)** |  |  |
| Right | 0.71 (0.18) | 0.76 (0.21) |
| Left | 0.78 (0.21) | 0.79 (0.30) |
| **Race — no. (%)** |  |  |
| White | 75 (93.8%) | 73 (91.3%) |
| Asian | 3 (3.8%) | 2 (2.5%) |
| Black | 1 (1.3%) | 3 (3.8%) |
| Other | 1 (1.3%) | 2 (2.5%) |
| **Medical history — no. (%)** |  |  |
| Hypertension | 56 (70.0%) | 50 (62.5%) |
| Stroke | 8 (10.0%) | 7 (8.8%) |
| Myocardial infarction | 12 (15.0%) | 14 (17.5%) |
| Hypercholesterolemia | 59 (73.8%) | 52 (65.0%) |
| Angina | 9 (11.3%) | 9 (11.3%) |
| Diabetes | 20 (25.0%) | 21 (26.3%) |
| Bypass revascularisation | 5 (6.3%) | 10 (12.5%) |
| Angio revascularisation | 13 (16.3%) | 23 (28.7%) |
| **Medication — no. (%)** |  |  |
| Antiplatelets | 65 (81.3%) | 67 (83.8%) |
| Glycoprotein IIb IIIa antagonists | 80 (100.0%) | 80 (100.0%) |
| Statin | 70 (87.5%) | 64 (80.0%) |
| Anticoagulant | 10 (12.5%) | 10 (12.5%) |
| Antihypertensives | 58 (72.5%) | 52 (65.0%) |

* Plus–minus values are means ±SD. No significant differences were identified between the treatment groups in any baseline variable. Percentages may not total 100 because of rounding.

#

**eTable 6. Output of the right censored^1^ multilevel Tobit model to assess the effects of baseline characteristics for ICD** at 3, 6, and 12 months for the ITT population (N=159)**

|  | **Multilevel Tobit Model  (ICD Square Root transformation)** |
| --- | --- |
|  | Model 1 |
| **Square root of the ICD at baseline** | 0.64 [0.47,0.81] p<0.001 |
| **Treatment^2^** |  |
| Control: BMT and BMT+SET |  |
| Treatment: NMES + BMT and NMES+BMT+SET | 0.97 [-0.6,2.55] p=0.23 |
| **Time^3^** |  |
| Month 3 |  |
| Month 6 | 0.83 [-0.23,1.88] p=0.12 |
| Month 12 | 1.79 [0.71,2.86] p<0.001 |
| **Treatment *Time^4^** |  |
| Treatment: NMES + BMT and NMES+BMT+SET*Month 6 | -0.41 [-1.89,1.06] p=0.58 |
| Treatment: NMES + BMT and NMES+BMT+SET*Month 12 | -0.4 [-2.02,1.21] p=0.63 |
| **Type of centre^5^** |  |
| Non-SET |  |
| SET | 2.33 [0.91,3.76] p<0.001 |
| **Age** | 0.01 [-0.07,0.09] p=0.84 |
| **Gender^6^** |  |
| Female |  |
| Male | -1.83 [-3.45,-0.22] p=0.03 |
| **BMI** | 0.03 [-0.09,0.14] p=0.65 |
| **Smoking^7^** |  |
| Never |  |
| Current smoker | -0.22 [-3.4,2.97] p=0.89 |
| Former smoker | 1.09 [-1.86,4.03] p=0.47 |
| **constant** | 4.43 [-4.03,12.89] p=0.31 |

Number of obs = 402, uncensored =388, Right-censored =14, Number of groups = 159

Multilevel Tobit model: square root of Initial Claudication distance (3, 6 and 12 months) = intercept +square root of ICD(Baseline) + Treatment + Time + interaction of Treatment and Time + Type of center + Age +Gender +BMI +Smoking Status

**The Square root transformation of AWD was used for baseline, 3 months, 6 months and 12 months measurements. Square root transformation variables satisfy the assumptions of the Multilevel Tobit model

^1^Right censoring set up at square root of AWD at 790 m (28.106939)

^2^Control: Local Available Exercise Therapy (BMT and BMT+SET) as reference category

^3^Month 3 as reference category

^4^Control and Month 3 as reference category for the interaction term (treatment and time)

^5^Non-SET exercise centers as reference category

^6^Female as reference category

^7^Never smoked as reference standard

**eTable 7. Output of linear mixed model^1^ for changes in the transformed log Right Ankle Brachial Pressure Index (ABPI)* between baseline and follow up periods (3, 6 and 12 months) for the ITT population N=159**

|  | **Log Right Ankle Brachial Pressure Index (ABPI)** |
| --- | --- |
|  | Coefficient [95% CI] p-value |
| **Log Right Ankle ABPI at baseline^1^** | 0.82 [0.71,0.93] p<0.001 |
| **Treatment^2^** |  |
| Control: EA and EA+SET |  |
| Treatment: NMES + EA and NMES+EA+SET | 0.05 [-0.02,0.12] p=0.17 |
| **Time^3^** |  |
| Month 3 |  |
| Month 6 | 0.07 [0.02,0.12] p=0.01 |
| Month 12 | 0.07 [0.02,0.13] p=0.01 |
| **Treatment*Time^4^** |  |
| Treatment: NMES + EA and NMES+EA+SET*Month 6 | -0.04 [-0.11,0.04] p=0.34 |
| Treatment: NMES + EA and NMES+EA+SET*Month 12 | -0.06 [-0.13,0.02] p=0.16 |
| **Constant** | -0.12 [-0.18,-0.06] p<0.001 |

*The log Right ABPI variables satisfy the assumptions of the Linear Mixed models.

^1^Mixed model adjusted for log Right Index (ABPI) at baseline, with time, treatment, and interaction term time and treatment as fixed effects and patient as random effect.

^2^Control: Local Available Exercise Therapy (BMT and BMT+SET) as reference category

^3^3 months as reference category

^4^Control and 3 months as reference category for the interaction term (treatment and time)

**eTable 8. Output of linear mixed model^1^ for changes in the transformed log Left Ankle Brachial Pressure Index (ABPI)* between baseline and follow up periods (3, 6 and 12 months) for the ITT population N=160**

|  | **Log Left Ankle Brachial Pressure Index (ABPI)** |
| --- | --- |
|  | Coefficient [95% CI] p-value |
| **Log left Ankle ABPI at baseline^1^** | 0.75 [0.66,0.83] p<0.001 |
| **Treatment^2^** |  |
| Control: EA and EA+SET |  |
| Treatment: NMES + EA and NMES+EA+SET | -0.03 [-0.1,0.03] p=0.32 |
| **Time^3^** |  |
| Month 3 |  |
| Month 6 | 0.002 [-0.06,0.07] p=0.93 |
| Month 12 | -0.02 [-0.08,0.05] p=0.59 |
| **Treatment*Time^4^** |  |
| Treatment: NMES + EA and NMES+EA+SET*Month 6 | 0.01 [-0.08,0.1] p=0.78 |
| Treatment: NMES + EA and NMES+EA+SET*Month 12 | 0.03 [-0.06,0.13] p=0.49 |
| **Constant** | -0.05 [-0.12,0.01] p=0.11 |

*The log Left ABPI variables satisfy the assumptions of the Linear Mixed models.

^1^Linear Mixed model adjusted for log Left Index (ABPI) at baseline, with time, treatment and interaction term time and treatment as fixed effects and patient as random effect.

^2^Control: Local Available Exercise Therapy (BMT and BMT+SET) as reference category

^3^3 months as reference category

^4^Control and 3 months as reference category for the interaction term (treatment and time)

**eTable 9. Summary of Quality-of-life outcomes – sub-domains of SF-36**

|  | **Baseline** | **3 months** | **6 months** | **12 months** |
| --- | --- | --- | --- | --- |
| **SF-36 Physical Function** |  |  |  |  |
| Treatment: NMES + BMT and NMES+BMT+SET  Control: BMT and BMT+SET  Difference† | 34.84 (8.47) [n = 91]  33.83 (8.34) [n = 97] | 37.93 (8.35) [n = 85]  35.25 (8.90) [n = 84]  1.5 (-0.6, 3.7) | 38.56 (9.59) [n = 79]  36.04 (9.65) [n = 78]  1.6 (-0.6, 3.8) | 37.60 (10.31) [n = 77]  37.78 (9.43) [n = 76]  -0.8 (-3, 1.5) |
| **SF-36 Role-Physical** |  |  |  |  |
| Treatment: NMES + BMT and NMES+BMT+SET  Control: BMT and BMT+SET  Difference† | 39.38 (11.20) [n = 91]  39.74 (11.64) [n = 96] | 43.68 (11.52) [n = 85]  40.95 (11.85) [n = 84]  3.3 (0, 6.6) | 43.53 (12.53) [n = 79]  40.71 (12.06) [n = 78]  3.3 (-0.1, 6.7) | 41.07 (11.82) [n = 76]  41.81 (12.11) [n = 75]  0.3 (-3.2, 3.7) |
| **SF-36 Body Pain** |  |  |  |  |
| Treatment: NMES + BMT and NMES+BMT+SET | 40.82 (9.52) [n = 91] | 43.04 (9.19) [n = 85] | 44.24 (10.37) [n = 79] | 43.56 (10.63) [n = 76] |
| Control: BMT and BMT+SET | 40.32 (8.57) [n = 96] | 40.48 (9.21) [n = 84] | 42.02 (10.04) [n = 78] | 43.76 (10.17) [n = 75] |
| Difference† |  | 1.2 (-1.5, 3.8) | 1.2 (-1.5, 4) | -1.1 (-3.9, 1.7) |
| **SF-36 General Health** |  |  |  |  |
| Treatment: NMES + BMT and NMES+BMT+SET | 42.25 (9.63) [n = 91] | 42.66 (9.96) [n = 85] | 43.17 (9.53) [n = 79] | 42.60 (11.64) [n = 77] |
| Control: BMT and BMT+SET | 42.50 (9.45) [n = 97] | 41.63 (10.11) [n = 84] | 41.06 (11.16) [n = 78] | 42.29 (10.12) [n = 76] |
| Difference† |  | 0.7 (-1.4, 2.9) | 1.2 (-0.97, 3.4) | 0.1 (-2.1, 2.3) |
| **SF-36 Vitality** |  |  |  |  |
| Treatment: NMES + BMT and NMES+BMT+SET | 47.02 (9.75) [n = 91] | 49.39 (8.19) [n = 84] | 48.96 (9.75) [n = 79] | 49.70 (10.43) [n = 77] |
| Control: BMT and BMT+SET | 46.27 (10.52) [n = 96] | 45.90 (10.83) [n = 84] | 46.32 (9.47) [n = 77] | 47.65 (9.93) [n = 76] |
| Difference† |  | 2.5 (-0.1, 5) | 1.8 (-0.8, 4.4) | 1.3 (-1.3, 3.9) |
| **SF-36 Social Functioning** |  |  |  |  |
| Treatment: NMES + BMT and NMES+BMT+SET | 45.09 (11.50) [n = 91] | 46.66 (10.88) [n = 85] | 47.31 (11.57) [n = 79] | 44.94 (13.10) [n = 77] |
| Control: BMT and BMT+SET | 42.81 (12.59) [n = 97] | 43.31 (12.25) [n = 84] | 44.19 (11.58) [n = 78] | 44.40 (11.89) [n = 75] |
| Difference† |  | 0.8 (-2.4, 4) | 0.8 (-2.4, 4.1) | 1.3 (-1.3, 3.9) |
| **SF-36 Role-Emotional** |  |  |  |  |
| Treatment: NMES + BMT and NMES+BMT+SET | 45.39 (12.98) [n = 91] | 46.79 (12.43) [n = 85] | 47.14 (12.18) [n = 79] | 45.78 (13.01) [n = 76] |
| Control: BMT and BMT+SET | 43.27 (13.84) [n = 96] | 42.55 (14.45) [n = 84] | 43.66 (13.86) [n = 78] | 43.96 (12.93) [n = 75] |
| Difference† |  | 2.2 (-1.9, 6.3) | 1.3 (-2.9, 5.4) | 0.6(-3.6, 4.8) |
| **SF-36 Mental Health** |  |  |  |  |
| Treatment: NMES + BMT and NMES+BMT+SET | 50.57 (10.62) [n = 91) | 52.21 (9.17) [n = 84] | 51.86 (10.15) [n = 79] | 52.10 (11.12) [n = 77] |
| Control: BMT and BMT+SET | 48.34 (11.48) [n = 96] | 47.27 (12.03) [n = 84] | 47.77 (11.44) [n = 77] | 48.31 (11.70) [n = 76] |
| Difference† |  | 1.8 (-0.9, 4.5) | 1 (-1.7, 3.8) | 1.3 (-1.5, 4.1) |

† The between-group differences were estimated by a mixed model adjusted for each baseline quality of life score and time as fixed effects and center and patients as random effects. The control group was the reference group. The widths of the confidence intervals were not adjusted for multiple comparisons and should not be used for formal reference.

‖ Scores on the Medical Outcomes Study 36-Item Short-Form Health Survey (SF-36) Summary range from 1 to 100, with higher scores indicating better quality of life.

**eTable 10. Serious adverse events – overall population by as treated**

| **Variable** | **Treatment: NMES + BMT and NMES+BMT+SET** | **Control: BMT and BMT+SET** | **Total** |
| --- | --- | --- | --- |
|  | **N=13** | **N=16** | **N=29** |
| **Severity N (%)** |  |  |  |
| Mild | 1 (7.7%) | 0 (0.0%) | 1 (3.4%) |
| Moderate | 2 (15.4%) | 4 (25.0%) | 6 (20.7%) |
| Severe | 7 (53.8%) | 6 (37.5%) | 13 (44.8%) |
| Life threatening or disabling | 1 (7.7%) | 5 (31.3%) | 6 (20.7%) |
| Fatal | 2 (15.4%) | 1 (6.3%) | 3 (10.3%) |
| **Outcome N (%)** |  |  |  |
| Recovered | 10 (76.9%) | 13 (81.3%) | 23 (79.3%) |
| Recovering/Improving | 1 (7.7%) | 0 (0.0%) | 1 (3.4%) |
| Not recovered | 0 (0.0%) | 1 (6.3%) | 1 (3.4%) |
| Fatal | 2 (15.4%) | 2 (12.5%) | 4 (13.8%) |
| Not assessable | 0 (0.0%) | 0 (0.0%) | 0 (0.0%) |
| **Causal Relationship to device N (%)** |  |  |  |
| Definitely | 0 (0.0%) | 0 (0.0%) | 0 (0.0%) |
| Probably | 0 (0.0%) | 0 (0.0%) | 0 (0.0%) |
| Possibly | 0 (0.0%) | 0 (0.0%) | 0 (0.0%) |
| Unlikely | 2 (15.4%) | 1 (6.3%) | 3 (10.3%) |
| Not related | 10 (76.9%) | 15 (93.8%) | 25 (86.2%) |
| Not assessable | 0 (0.0%) | 0 (0.0%) | 0 (0.0%) |
| Not Applicable | 1 (7.7%) | 0 (0.0%) | 1 (3.4%) |

**eTable 11. Descriptive statistics of Absolute Walking Distance (AWD) at baseline**

| **Variable** | **N** | **Mean** | **SD** | **p25** | **p50** | **p75** | **Min** | **Max** |
| --- | --- | --- | --- | --- | --- | --- | --- | --- |
| AWD at Baseline | 190 | 231.1848 | 169.0166 | 100 | 175.735 | 340 | 1.27 | 756.39 |

**eTable 12. Stratification of Absolute Walking Distance (AWD) by low, medium, and high distance**

|  | **Patients** | **Pct** |
| --- | --- | --- |
| Short - Less than 100 m | 48 | 25% |
| Medium - Between 100 and 340 m | 98 | 52% |
| Long - More than 340 m | 44 | 23% |
| **Total** | 190 | 100% |

**eTable 13. Output of the right censored^1^ Tobit regression model^2^ for Absolute Walking Distance (AWD)** at 3 months for patients who walked a short distance at baseline in the ITT population (N=40)**

|  | **Tobit regression  (AWD Square Root transformation)** |
| --- | --- |
|  | **Model 1** |
| **Square root of AWD at baseline** | 1.18 [0.26,2.09] p=0.01 |
| **Treatment^3^** |  |
| Control: BMT and BMT+SET |  |
| Treatment: NMES + BMT and NMES+BMT+SET | 0.43 [-3.5,4.35] p=0.83 |
| **Type of centre^4^** |  |
| Non-SET |  |
| SET | 2.93 [-1.02,6.87] p=0.14 |
| **constant** | 2 [-6.61,10.61] p=0.64 |

**The Square root transformation of AWD was used for baseline and 3 months measurements. Square root transformation variables satisfy the assumptions of the Tobit model

^1^Right censoring set up at (28.106939) square root of AWD at 790 m

^2^Tobit Regression model: square root of AWD at 3 months = intercept + square root of AWD (baseline) + Treatment +Type of center

^3^Control: Local Available Exercise Therapy (BMT and BMT+SET) as reference category

^4^Non-SET exercise centers as reference category

**eTable 14. Output of the right censored^1^ Tobit regression model^2^ for Absolute Walking Distance (AWD)** at 3 months for patients who walked a medium distance at baseline in the ITT population (N=80)**

|  | **Tobit regression  (AWD Square Root transformation)** |
| --- | --- |
|  | Model 1 |
| **Square root of AWD at baseline** | 0.87 [0.42,1.32] p<0.001 |
| **Treatment^3^** |  |
| Control: BMT and BMT+SET |  |
| Treatment: NMES + BMT and NMES+BMT+SET | 0.22 [-1.81,2.24] p=0.83 |
| **Type of centre^4^** |  |
| Non-SET |  |
| SET | 3.08 [1.03,5.14] p=0 |
| **constant** | 2.99 [-3.72,9.7] p=0.38 |

**The Square root transformation of AWD was used for baseline and 3 months measurements. Square root transformation variables satisfy the assumptions of the Tobit model

^1^Right censoring set up at (28.106939) square root of AWD at 790 m

^2^Tobit Regression model: square root of AWD at 3 months = intercept + square root of AWD (baseline) + Treatment +Type of center

^3^Control: Local Available Exercise Therapy (BMT and BMT+SET) as reference category

^4^Non-SET exercise centers as reference category

**eTable 15. Output of the right censored^1^ Tobit regression model^2^ for Absolute Walking Distance (AWD)** at 3 months for patients who walked a long distance at baseline in the ITT population (N=40)**

|  | **Tobit regression  (AWD Square Root transformation)** |
| --- | --- |
|  | Model 1 |
| **Square root of AWD at baseline** | 0.82 [0.36,1.28] p<0.001 |
| **Treatment^3^** |  |
| Control: BMT and BMT+SET |  |
| Treatment: NMES + BMT and NMES+BMT+SET | 2.88 [0.51,5.25] p=0.02 |
| **Type of centre^4^** |  |
| Non-SET |  |
| SET | 4.03 [1.61,6.45] p<0.001 |
| **constant** | 1.48 [-8.81,11.77] p=0.77 |

**The Square root transformation of AWD was used for baseline and 3 months measurements. Square root transformation variables satisfy the assumptions of the Tobit model

^1^Right censoring set up at (28.106939) square root of AWD at 790 m

^2^Tobit Regression model: square root of AWD at 3 months = intercept + square root of AWD (baseline) + Treatment +Type of center

^3^Control: Local Available Exercise Therapy (BMT and BMT+SET) as reference category

^4^NonSET exercise centers as reference category

**References**

**eReferences**

1. Devlin, N.J., et al., *Valuing health-related quality of life: An EQ-5D-5L value set for England.* Health Economics, 2018. 27(1): p. 7-22.

2. Garratt, A.M., et al., *Responsiveness of the SF-36 and a condition-specific measure of health for patients with varicose veins.* Qual Life Res, 1996. 5(2): p. 223-34.

3. Chong, P.F., et al., *The intermittent claudication questionnaire: a patient-assessed condition-specific health outcome measure.* J Vasc Surg, 2002. 36(4): p. 764-71; discussion 863-4.
